# Separation of Palladium from Alkaline Cyanide Solutions through Microemulsion Extraction Using Imidazolium Ionic Liquids

**DOI:** 10.3390/ijms241310709

**Published:** 2023-06-27

**Authors:** Hui Deng, Chali Liu, Xin Xu, Yuanyuan Wu, Muhan Chen, Zhangjie Huang

**Affiliations:** School of Chemical Science and Technology, Yunnan University, Kunming 650091, China; 15181601384@163.com (H.D.); fbg@mail.ynu.edu.cn (C.L.); 12022116032@mail.ynu.edu.cn (X.X.); 12022116035@mail.ynu.edu.cn (Y.W.)

**Keywords:** microemulsions, ionic liquids, metal cyanides, palladium

## Abstract

In this paper, three imidazolium-based ionic liquids, viz., 1-butyl-3-undecyl imidazolium bromide ([BUIm]Br), 1-butyl-3-octyl imidazolium bromide ([BOIm]Br), and 1-butyl-3-hexadecyl imidazolium bromide ([BCIm]Br), were synthesized. Three novel microemulsions systems were constructed and then were used to recover Pd (II) from cyanide media. Key extraction parameters such as the concentration of ionic liquids (ILs), equilibration time, phase ratio (R_A/O_), and pH were evaluated. The [BUIm]Br/n-heptane/n-pentanol/sodium chloride microemulsion system exhibited a higher extraction percentage of Pd (II) than the [BOIm]Br/n-heptane/n-pentanol/sodium chloride and [BCIm]Br/n-heptane/n-pentanol/sodium chloride microemulsion systems. Under the optimal conditions (equilibrium time of 10 min and pH 10), the extraction percentages of these metals were all higher than 98.5% when using the [BUIm]Br/n-heptane/n-pentanol/sodium chloride microemulsion system. Pd(CN)_4_^2−^ was separated through a two-step stripping procedure, in which Fe (III) and Co (III) were first separated using KCl solution, then Pd(CN)_4_^2−^ was stripped using KSCN solution (separation factors of Pd from Fe and Co exceeded 10^3^). After five extraction–recovery experiments, the recovery of Pd (II) through the microemulsion system remained over 90%. The Pd (II) extraction mechanism of the ionic liquid [BUIm]Br was determined to occur via anion exchange, as shown by spectral analysis (UV, FTIR), Job’s method, and DFT calculations. The proposed process has potential applications for the comprehensive treatment of cyanide metallurgical wastewater.

## 1. Introduction

Due to its catalytic activity, electrical conductivity, and high temperature stability, palladium (Pd) is widely used in biomedical devices, electronics equipment, automotive catalytic converters, and jewelry [1,2,3,4]. Jinbao Mountain in western Yunnan is the main producer of platinum group metals (PGMs) in China, which are mainly extracted from minerals through pressurized cyanidation. The wastewater of the tailing ponds of mines contains large amounts of metal cyanides [5,6], which release CN^−^ under certain conditions [7,8]. Therefore, to eliminate the harm to the environment, metal cyanide wastewater must be treated before it can be discharged. In addition, Jinbao Mountain’s metallurgical wastewater also contains a large amount of palladium, which needs to be recovered before the wastewater can be discharged into the environment [9,10]. The disposal of metallic cyanide and the rescue process of metal ions from metallurgical wastewater occupy a vital position in environmental protection and the comprehensive development and utilization of mine resources.

The removal methods of metal cyanides include biodegradation methods [11,12], oxidation methods [13,14], ion exchange methods [15,16], and activated carbon adsorption methods [17,18]. Due to their high stability, metal cyanides have a slow biodegradation rate, and many steps are required to extract valuable metals [19,20]. Because the chemical reagents that are used harm the environment, oxidation methods create complex by-products that are difficult to treat. They also produce environmental pollution and have a narrow application range [21,22]. The ion exchange and activated carbon adsorption methods have low treatment capacities, poor selectivity, and poor regeneration [23,24]. These disadvantages of the above processes make them unsuitable for large-scale metal cyanide removal. By comparison, solvent extraction can be used to remove metal cyanides and recover valuable metals due to its large processing capacity and high selectivity [25,26,27].

Quaternary ammonium salts, quaternary phosphonium salts, and imidazolium-based ionic liquid extraction systems have been used to remove metal cyanides from metallurgical wastewater and recover valuable metals such as gold [28], platinum, and palladium [5] due to their thermal stability, low toxicity, insolubility, structural tunability, and low volatility [29,30,31,32]. Jin C et al. [33] used sulfonated kerosene as a diluent and three phosphonium-based ILs as extractants to remove metal cyanides and recover palladium from metallurgical wastewater. Under the optimal conditions, the phosphonium-based IL TOUPB has good extraction ability for Pd (II). However, ILs have high viscosity, easily emulsify, and are difficult to remove. Lu [34] constructed a CTAB/n-heptane/isopentyl alcohol/Na_2_SO_3_ microemulsion system and investigated the parameters affecting the Au (III) extraction from a hydrochloric acid medium. The results showed that Au (III) can be extracted selectively and effectively. Yu [35] explored the parameters affecting the extraction rate of Au (III) by formulating a microemulsion system using the IL [C_14_mim]Br with n-hexanol, cyclohexane, and hydrochloric acid. The organic phase containing Au (III) was stripped by oxalic acid to obtain Au (0). The use of ionic liquid microemulsions, which combine the benefits of ILs and microemulsions, will expand the application range of solvent extraction. They have been extensively used in palladium extraction and separation. Zheng [36] constructed an organosilicon IL [Si_4_mim]Cl microemulsion system for Pd (II) extraction from hydrochloric acid media. The effects of key parameters such as oscillation time, phase ratio (R_A/O_), and [Si_4_mim] Cl concentration were explored, and the extraction rate exceeded 98% under the optimal conditions. Finally, to strip the organic phase with Pd (II), a high concentration of NaCl was applied. Nguyen et al. [37] constructed an IL101/TX100/water microemulsion and used a five-step separation scheme to extract gold, palladium, and platinum from hydrochloric acid media. There were many time-consuming steps, and acidic thiourea was used to strip the organic phase containing palladium and platinum. Shao M et al. [38] constructed a [C8Y3Pr]Br microemulsion system to extract Pd (II). The organic phase containing Pd (II) was reduced using alcohol to obtain Pd nanoparticles, and a microemulsion was regenerated. These microemulsion systems have only been used in acidic media, while palladium separation and extraction from alkaline media using ionic liquid microemulsion systems have not been reported in the literature.

To develop an ionic liquid–microemulsion extraction system to extract and separate PGMs in alkaline cyanide liquid media, 1-butyl-3-undecyl imidazolium ionic liquid was used as an extractant with n-pentanol as a co-surfactant, and a [BUIm]Br/n-heptane/n-pentanol/NaCl microemulsion system was constructed to remove mixed metal cyanides and to recover palladium. The effects of key extraction parameters such as time, [BUIm]Br concentration, and pH were studied. Pd (II) extraction by the [BUIm]Br/n-heptane/n-pentanol/sodium chloride microemulsion system was confirmed through UV and FTIR spectroscopic analysis and the continuous variation method (Job’s method). DFT calculations were used to determine the ion exchange mechanism. Pd (II) in the microemulsion was stripped using KSCN, the organic phase was regenerated, and the microemulsion was reused.

## 2. Results and Discussion

### 2.1. Extraction Behavior

#### 2.1.1. Influence of Oscillation Time

**Figure 1:**
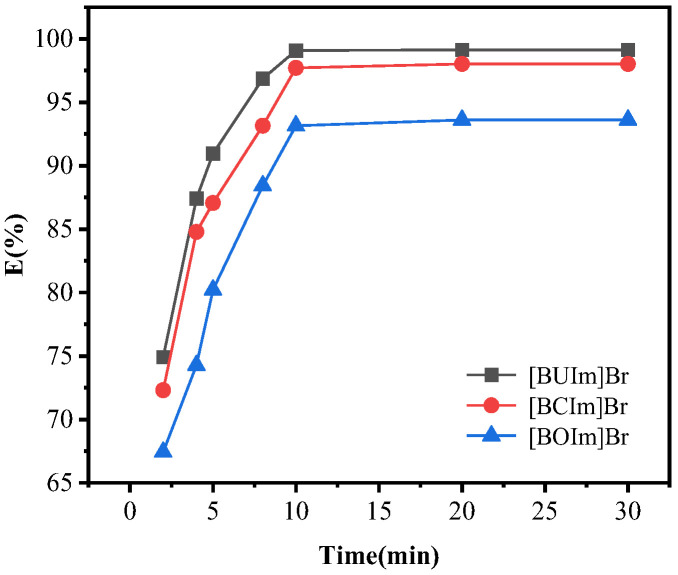
Influence of oscillation time. C_Pd (II)_ = 0.5 mM, R_A/O_ = 1, pH = 10, t = 10 min, T = 298.15 K, C_ILs_ = 0.05 M.

To explore the influence of oscillation time on extraction performance, the oscillation time was changed from 2 min to 30 min, and the other extraction parameters were fixed. The extraction rate increased and reached equilibrium at 10 min (Figure 1). Therefore, an equilibration time of 10 min was sufficient for complete extraction.

#### 2.1.2. Influence of Ionic Liquid Concentration

**Figure 2:**
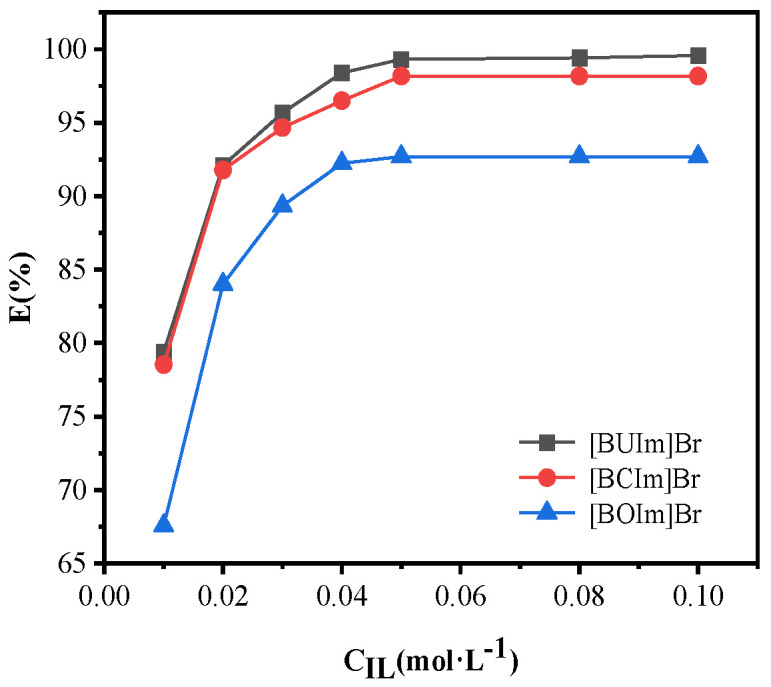
Influence of ILs concentration. C_Pd (II)_ = 0.5 mM, R_A/O_ = 1, pH = 10, t = 10 min, T = 298.15 K, C_ILs_ = 0.05 M.

The concentration of the three ionic liquids for palladium extraction was changed from 0.01 M to 0.1 M. The water phase contained 0.5 mM Pd (II), pH 10, R_A/O_ = 1, and oscillation for 10 min. As shown in Figure 2, at an ionic liquid concentration of 0.05 M, the extraction rates of [BUIm]Br, [BCIm]Br, and [BOIm]Br were 99.5%, 98.2%, and 92.7%, respectively. With the ILs concentration increased from 0.01 M to 0.05 M, the extraction rate of Pd (II) rapidly increased. With a further increase in the concentration of the ILs from 0.05M to 0.1M, the Pd (II) extraction rate remained essentially unchanged. Therefore, 0.05M ILs were employed in extracting Pd (II). Figure 2 showed that at the same ionic liquid concentration, the extraction rate was [BUIm]Br > [BCIm]Br > [BOIm]Br.

#### 2.1.3. Influence of pH

**Figure 3:**
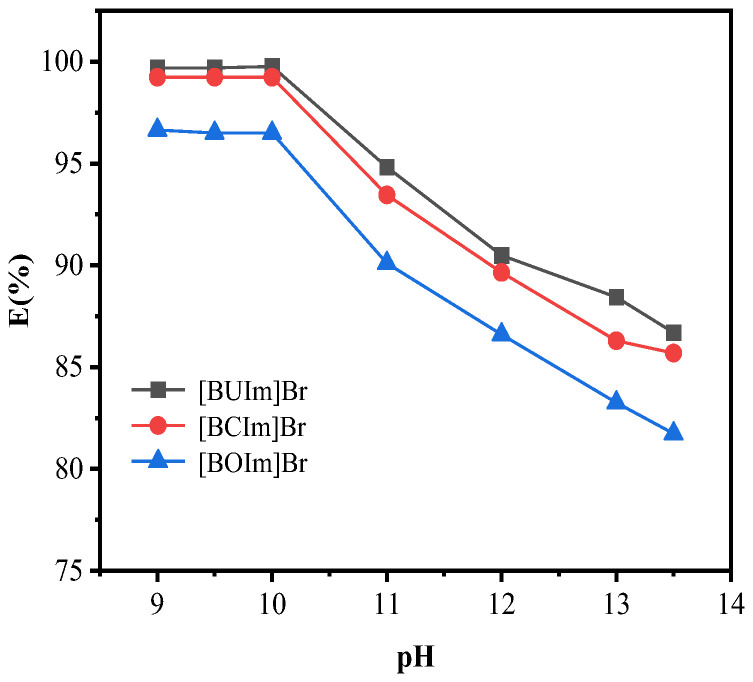
Influence of pH of the water phase. C_Pd (II)_ = 0.5 mM, R_A/O_ = 1, pH = 10, t = 10 min, T = 298.15 K, C_ILs_ = 0.05 M.

The extraction of Pd (II) is affected by the pH of the solution. The pH of the water phase was changed from 9 to 14, while the other experimental conditions remained the same. The experimental results are shown in Figure 3. When pH < 10, the extraction rate remained constant, and the highest extraction rates were observed. When pH > 10, the extraction rate of palladium decreased. The low extraction rate at a high pH may be due to the competition with OH^−^ [39]. Therefore, the optimal condition was an aqueous phase pH of 10.

#### 2.1.4. Influence of the Phase Ratio (Water/Organic)

**Figure 4:**
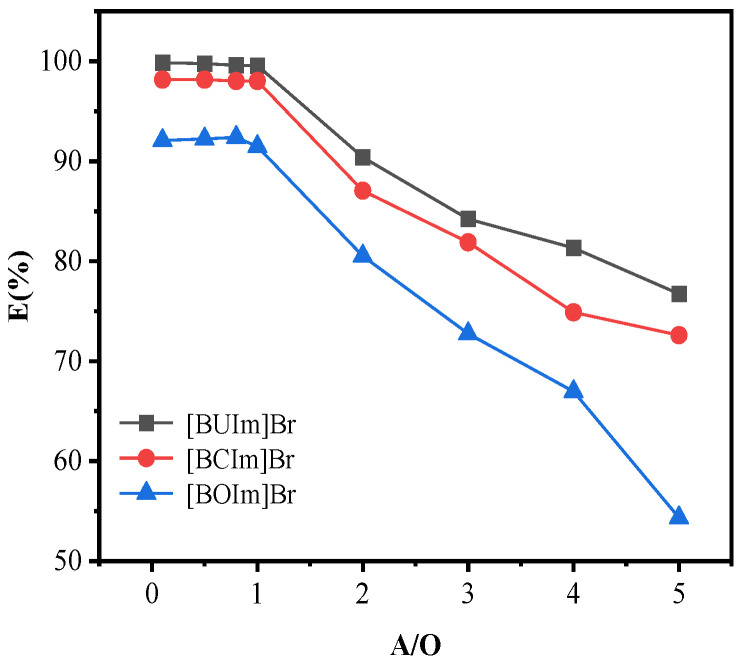
Influence of the phase ratio. C_Pd (II)_ = 0.5 mM, R_A/O_ = 1, pH = 10, t = 10 min, T = 298.15 K, C_ILs_ = 0.05 M.

Keeping other conditions consistent, the effect of the phase ratio was examined. The R_A/O_ varied from 0.1 to 5. The results in Figure 4 show that the extraction rate was highest and remained constant when the ratio was less than 1. However, the Pd (II) extraction percentage decreased upon further increasing the phase ratio, so R_A/O_ = 1 removed Pd (II). The concentration of the extractant in the organic phase is constant, so the extraction capacity is stationary. As the R_A/O_ increases, the extracted substance in the aqueous phase increases, resulting in a decrease in the extraction rate [40].

#### 2.1.5. Influence of NaBr Concentration

**Figure 5:**
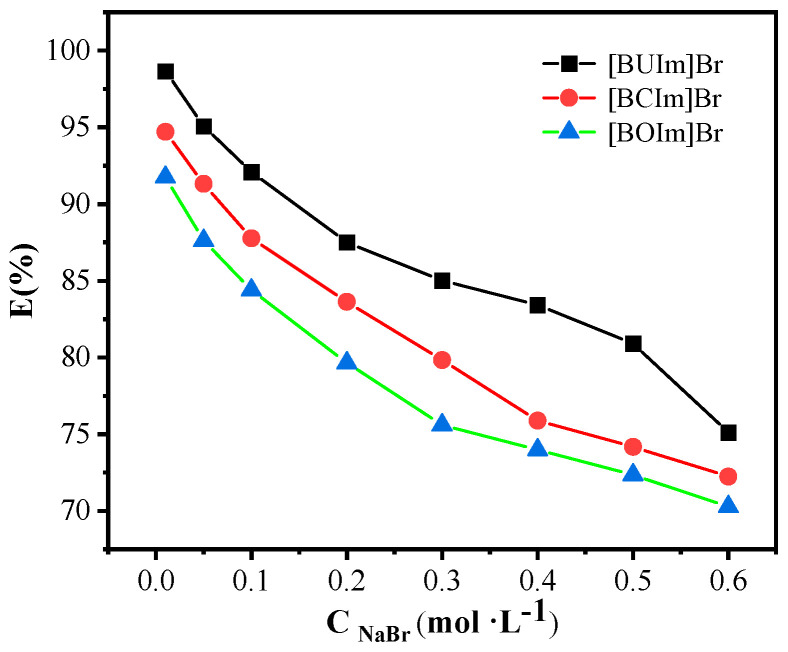
Influence of NaBr concentration on extraction Pd (II). Microemulsion phase: C_ILs_ = 0.05 M; Aqueous phase: C _Pd (II)_ = 0.5 mM, R_A/O_ = 1, pH = 10, t = 10 min, T = 298.15 K.

The effect of NaBr concentration on the extraction of Pd (II) is shown in Figure 5. As can be seen from Figure 5, the extraction rate of Pd (II) markedly decreased with increasing the NaBr concentration from 0.01 M to 0.6 M. The experimental results indicate that the extraction process involves an anion exchange mechanism [33].

#### 2.1.6. Stripping of Palladium

Stripping plays an important role in the recirculation of organic phases. Inorganic salts such as KSCN and potassium halide were investigated as stripping reagents and were mixed with the organic phase containing Pd (II) at a phase ratio of 5 and shaken for 10 min. The stripping rates of different stripping reagents for Pd (II) are shown in Table 1. KSCN achieved a 99.5% stripping rate of Pd (II), so it was chosen as the stripping reagent in subsequent experiments. The effect of different concentrations of KSCN on the stripping rate of Pd (II) was investigated (Figure S10). The stripping rate increased with the KSCN concentration and reached 99.5% at a concentration of 0.5 mol·L^−1^. As the concentration of KSCN continued to increase, the stripping rate remained constant, so 0.5 mol·L^−1^ KSCN was selected to ensure complete stripping.

#### 2.1.7. Effect of Stripping Extraction Time

The organic phase containing Pd (II) was stripped using 0.5 M KSCN at room temperature, and the stripping rates obtained at different times are shown in Figure S11. The stripping rate increased from 55% to 99% over an extraction time range of 1–10 min and remained constant when the stripping time was further increased. Therefore, equilibration with 0.5 M KSCN for 10 min was effective for stripping Pd.

#### 2.1.8. Extraction of Palladium from Mixed Metal Solution

The imidazolium -based ILs have a strong extraction capacity and will extract Pd (II) along with Fe (III) and Co (III). Stepwise elution is required to separate Pd (II) from a mixed metal solution containing Fe (III), Co (III), and Pd (II) (0.5 mM each). After extraction, an aqueous phase was obtained. Metal ions were first extracted using 1 M KCl, followed by 0.5 M KSCN. The two phases were then separated, and the concentration of metal ions was measured. The results are shown in Table 2.

The stripping rates of Pd (II), Co (III), and Fe (III) were 2.9%, 98.0%, and 97.8%, respectively. The distribution ratios (*D*) of Fe (III) and Co (III) were 0.0204 and 0.0225, and the separation factors were 1.23 × 10^3^ (K_Pd/Co_) and 1.16 × 10^3^ (K_Pd/Co_). The separation factors were all greater than 10^3^. The second step stripped 96.1% of Pd (II), while the remaining Fe (III) and Co (III) could not be further extracted by using the KSCN solution. The recovery rate (R%) of Pd (II) was greater than 95.0%. Therefore, a two-step stripping process was necessary to selectively recover Pd(CN)_4_^2−^.

#### 2.1.9. Reusability of [BUIm]Br/n-Heptane/n-Pentanol/NaCl Microemulsion System

**Figure 6:**
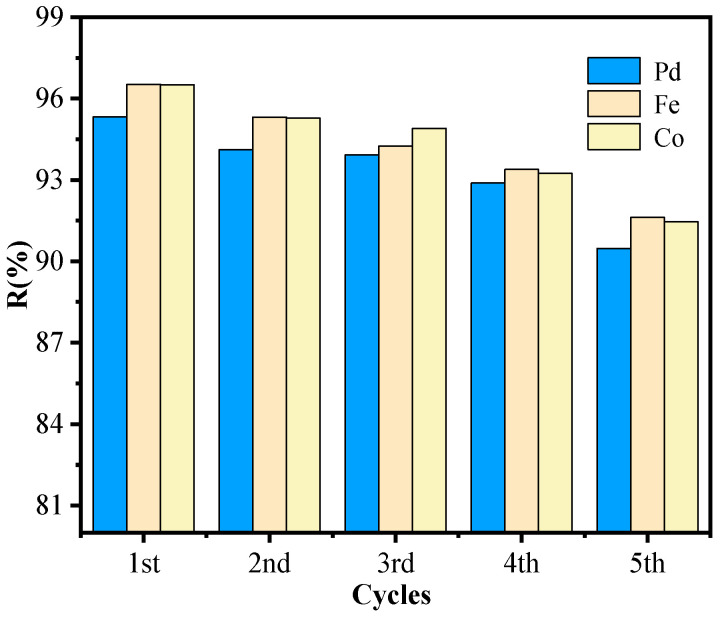
Extraction of Pd (II) in mixed metal solution.

The recoverability of the [BUIm]Br extractant was assessed to determine its reusability for commercial use. To evaluate this, a series of extraction–stripping experiments were carried out. After extraction, 0.5 mol·L^−1^ KSCN liquid was added, shaken for 10 min, and separated. The microemulsion phase was regenerated by using a 2 M potassium bromide solution. Extraction was continued by adding the solution to be extracted and repeating the process five times. After five extraction–stripping cycles in single Pd (II) solutions, the Pd (II) recovery rate remained above 90% (Table 3), demonstrating the reusability of the [BUIm]Br extraction system. To further confirm the reusability of the [BUIm]Br system extraction, the effect of the presence of iron and cobalt was also determined. After five successive extractions–stripping cycles, the recovery of Pd (II) remained over 90% in a mixed metal solution containing Pd (II), Fe (III), and Co (III) (Figure 6).

### 2.2. Mechanistic Analysis

#### 2.2.1. Conductivity of Microemulsions

**Figure 7:**
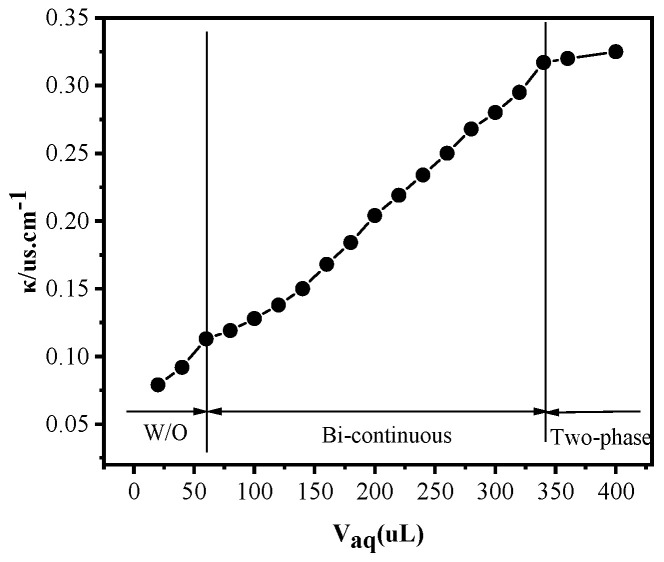
Variation in conductivity of [BUIm]Br/n-heptane/n-pentyl alcohol/NaCl microemulsions with volume of added water.

Water affects the structure of microemulsions, and electrical conductivity was used to explore the water content of the microemulsion to determine structural changes. For this, 0.01 M NaCl solution was added to the [BUIm]Br/n-heptane/pentanol system (30% of the total volume was pentanol), and the conductivity was measured with different water contents until the aqueous phase produced a precipitate. The results in Figure 7 show that when the water content of the system was low, the change in conductivity was small, which is typical of W/O-type microemulsions [41]. As the water content continued to increase, the microemulsion structure became bi-continuous, and the conductivity continued to increase [42,43]. As the volume of NaCl continued to increase, the organic phase began to delaminate, at which point, the microemulsion structure shifted to a non-homogeneous phase, and the conductivity remained constant.

#### 2.2.2. UV Spectra Analysis

**Figure 8:**
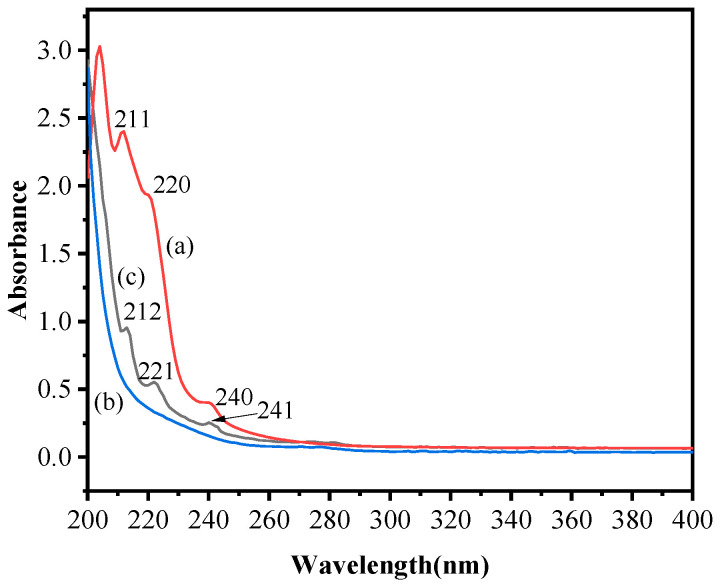
UV spectra of Pd(CN)_4_^2−^, [BUIm]Br, and [BUIm]- Pd (II). (**a**) Pd(CN)_4_^2−^, (**b**) [BUIm]Br, and (**c**) [BUIm]-Pd (II). C_Pd (II)_ = 0.5 mM, C _[BUIm]Br_ = 0.05 M.

UV spectra of [BUIm]Br, Pd(CN)_4_^2−^, and the extracted species were determined according to the methods described in the literature [5]. The characteristic UV spectra absorption peaks of Pd(CN)_4_^2−^ were located at 211 nm, 220 nm, and 240 nm, in agreement with the literature [33] (Figure 8a). The ionic liquid [BUIm]Br has no obvious UV absorption peak (Figure 8b). The characteristic peak of the extracted species ([BUIm]-Pd (II)) was similar to that of Pd(CN)_4_^2−^, with only a slight shift in the positions of the peaks due to different solvents (211 nm → 212 nm, 220 nm → 221 nm, and 240 nm → 241 nm) (Figure 8c). The UV spectra indicate that the extraction process of Pd (II) is based on the anion exchange mechanism.

#### 2.2.3. ^1^H NMR and Infrared Spectra Analysis

**Figure 9:**
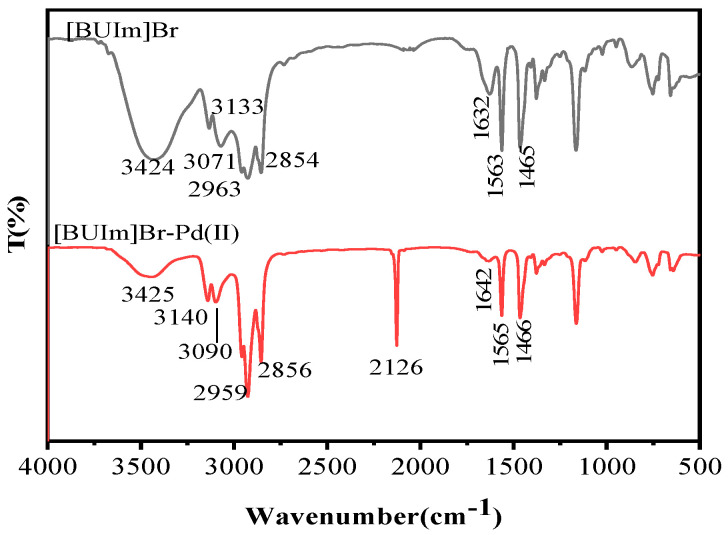
Infrared spectra of [BUIm]Br and [BUIm]-Pd (II) complexes.

To investigate the interaction mechanism between [BUIm]Br and Pd(CN)_4_^2−^, infrared spectra were used to analyze the [BUIm]-Pd (II) complexes (Figure 9), and absorption peaks are listed in Table 4. 

A comparison of the IR spectra of the [BUIm]-Pd (II) complexes shows that the absorption peaks associated with the imidazolium ring changed significantly: 3133 cm^−1^ → 3140 cm^−1^, 3071 cm^−1^ → 3140 cm^−1^, and 1632 cm^−1^ → 1642 cm^−1^. A new peak was observed at 2126 cm^−1^ [33] and was associated with the C≡N stretching vibration of Pd(CN)_4_^2^. The relatively weak interactions between Pd(CN)_4_^2−^ and the alkyl side chain caused the stretching peak of the aliphatic C–H groups to remain essentially unchanged. The results demonstrate interactions of Pd(CN)_4_^2^ with the [BUIm]Br cationic head group and weaker interactions with the alkyl side chain farther from the imidazolium ring.

**Figure 10:**
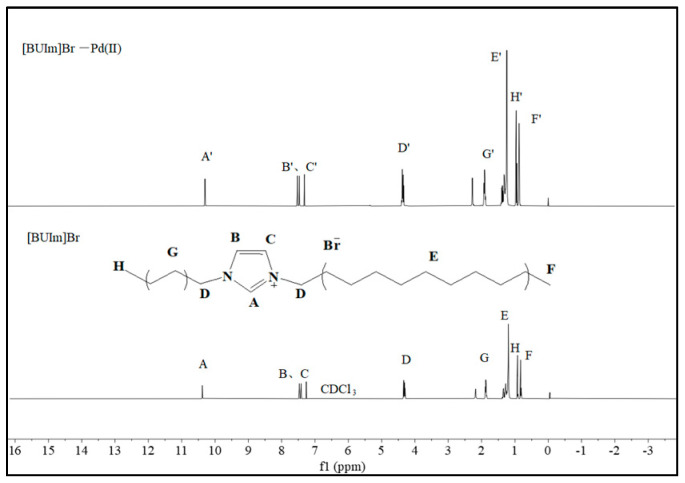
^1^H NMR spectra of [BUIm]Br and [BUIm]-Pd (II) complexes.

The [BUIm]Br and [BUIm]Br-Pd (II) complexes were examined through ^1^H NMR spectroscopy. The hydrogen nuclei (δH, ppm) shifted as follows: ΔδH near the imidazolium ring: A → A′, 10.38 → 10.24; B → B′, 7.47 → 7.55; C → C′, 7.42 → 7.47; D → D′, 4.32 → 4.37 (ΔδH ≥ 0.05). Farther away from the hydrogen imidazolium cation ΔδH: H → H′, 0.92 → 0.91; F → F′, 0.83 → 0.82 (ΔδH = 0.01) (Figure 10). The 1H NMR spectrum demonstrates the weak effect of the binding of [BUIm]Br to Pd(CN)_4_^2−^ on the hydrogen of the alkyl side chain in [BUIm]Br and the stronger effect on the hydrogen of the cationic [BUIm]^+^ head group.

#### 2.2.4. MS Analysis 

**Figure 11:**
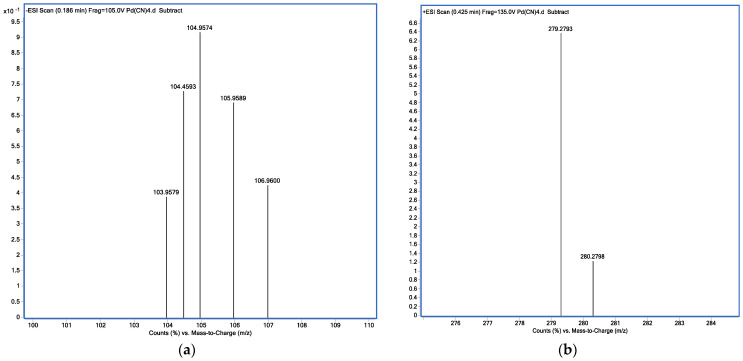
Mass spectrum of (**a**) negative ions (Pd(CN)_4_^2−^) and (**b**) positive ions ([BUIm]^+^).

The presence of palladium in the [BUIm]Br microemulsion phase was investigated. The negative ion peaks at m/z 104.9574, 104.4593, 103.9579, 105.9589, and 106.9600 were attributed to Pd(CN)_4_^2−^, demonstrating that the structure of palladium did not change during two-phase transfer (Figure 11a). A positive ion peak was found at m/z 279.2793 (C_18_H_35_N_2_^+^) (Figure 11b), proving that the cations were unchanged during extraction. This result provides further evidence that the extraction process occurred via ion exchange.

#### 2.2.5. Job’s Method

The effect of different extractive ratios of the [BUIm]Br-Pd (II) complexes was investigated through Job’s method [44]. The total amount of [BUIm]Br and Pd (II) were kept constant (3 × 10^−6^), pH 10, and the ratio of the amounts of [BUIm]Br to Pd (II) was varied from 0.5 to 2. It can be concluded from Figure S12 that the maximum extractive ratio of the two phases was 0.5, so the molar ratio of the Pd (II)/[BUIm]Br complexes was set at 1:2.

**Figure 12:**
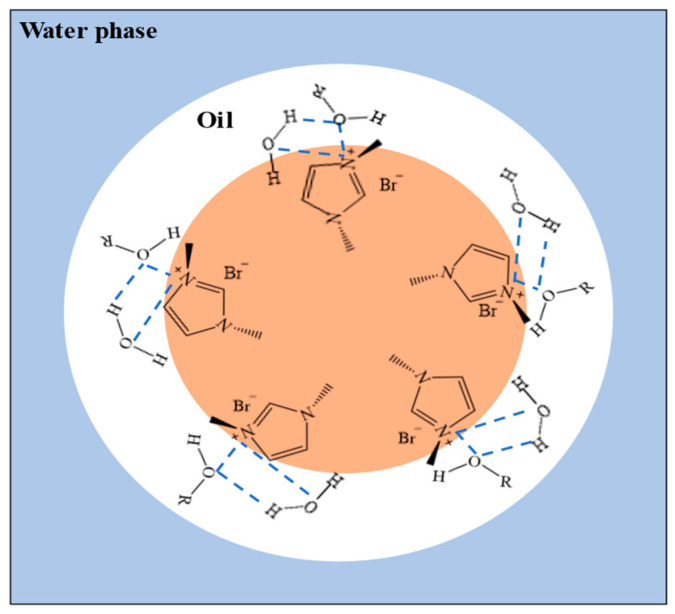
Structure of [BUIm]Br/n-heptane/n-pentanol/NaCl microemulsion during extraction.

Based on Job’s method and the spectrum of the [BUIm]Br-Pd (II) complex, it can be speculated that a 2:1 extractive complex was generated between [BUIm]Br and Pd (II) during extraction, while strong interactions existed between Pd(CN)_4_^2−^ and [BUIm]Br. Therefore, it was presumed that Pd (II) is extracted through anion exchange. Due to the addition of pentanol to the [BUIm]Br/n-heptane/n-pentanol/NaCl microemulsion system, other interactions can occur, such as ion–dipole interactions between n-pentanol and the imidazolium ring cation and hydrogen bonding between –OH and the N atom. These interactions will make the microemulsion system more stable and reduce mass loss during extraction [37,45]. The structure is shown in Figure 12. 

#### 2.2.6. Influence of Temperature 

**Figure 13:**
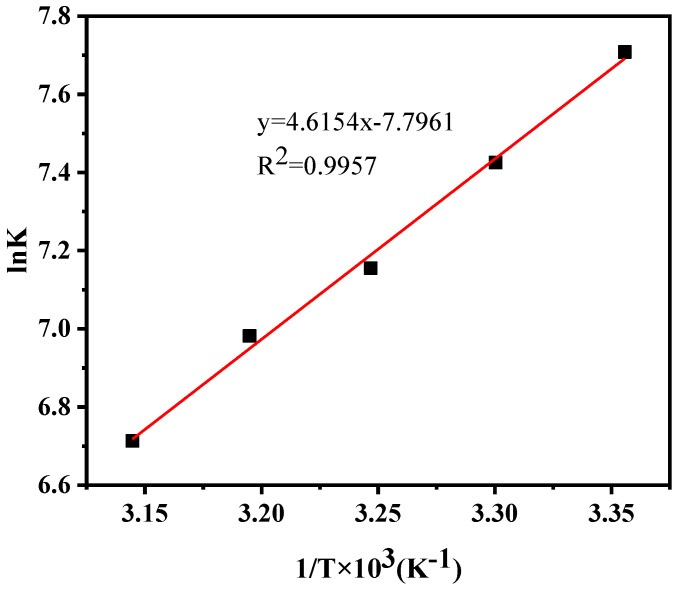
Influence of temperature on the extraction rate of Pd (II).

Changes in the palladium extraction rate were tested at different temperatures (298 K, 303 K, 308 K, 313 K, and 318 K). The experimental results (Figure 13) show that the Pd (II) extraction percentage gradually decreased upon increasing the temperature.

The ion exchange between [*BUIm*]*Br* and *Pd* (II) was proven by the previous spectral analysis:

(1) $$[Pd\left(CN{)}_{4}^{2-}\right]aq+2\left[BUIm^{+}Br^{-}\right]org=\;[{\left(BUIm^{+}\right)}_{2}Pd\left(CN{)}_{4}^{2-}]org+2\;[Br^{-}\right]aq$$

According to the ion exchange reaction equation, the partition ratio of *Pd* (II) in the microemulsion and water phase was calculated using Equation (2). The equilibrium constant of the reaction (*Ke*) was calculated using Equations (3) and (4).

(2) $$D=\frac{{\;[\mathrm{Pd}(\mathrm{CN})}_{4}^{2-}]\mathrm{org}}{{\;[\mathrm{Pd}(\mathrm{CN})}_{4}^{2-}]\mathrm{aq}}=\frac{{\;[(\mathrm{BUIm}}^{+}{)}_{2}{\mathrm{Pd}(\mathrm{CN})}_{4}^{2-}]\mathrm{org}}{{\;[\mathrm{Pd}(\mathrm{CN})}_{4}^{2-}]\mathrm{aq}}$$

(3) $$\mathrm{Ke}\left(T\right)=\frac{{\;[(\mathrm{BUIm}}^{+}{)}_{2}\mathrm{Pd}(CN{)}_{4}^{2-}{]\mathrm{org}\;[\mathrm{Br}}^{-}{]\mathrm{aq}}^{2}}{\left[{\mathrm{Pd}(\mathrm{CN})}_{4}^{2-}\right]\mathrm{aq}\left[{\mathrm{BUIm}}^{+}{\mathrm{Br}}^{-}\right]{\mathrm{org}}^{2}}$$

(4) $$\mathrm{Ke}\left(T\right)=D\cdot \frac{{\;[\mathrm{Br}}^{-}{]\mathrm{aq}}^{2}}{\left[{\mathrm{BUIm}}^{+}{\mathrm{Br}}^{-}\right]{\mathrm{org}}^{2}}\;$$

The enthalpy change of the reaction (∆*H*) was determined from the van’t Hoff diagram, and the Gibbs free energy (∆*G*) and entropy change (∆*S*) of the reaction were derived using thermodynamic Equations (5) and (6).

(5) $${\mathrm{lnK}}_{e}\left(T\right)=-\frac{\Delta H}{R}\cdot \frac{1}{T}+\frac{\Delta S}{R}\;\;$$

(6) ΔG=ΔH − TΔS

where *R* (8.314 J K^−1^ mol^−1^) in Equation (5) is the molar constant of the gas. The thermodynamic constants of the [BUIm]Br/heptane/pentanol/NaCl microemulsion system for the extraction process of *Pd* (II) are shown in Table 5. The results indicate that the reaction process is exothermic.

### 2.3. Density Functional Theory (DFT) Study

To identify extractant active sites and reveal interactions between the metal cyanide anions and imidazolate cations that occur at the interface of the microemulsion system, the geometry of the products [BOIm]^+^, [BUIm]^+^, and [BCIm]^+^ binding with Pd(CN)_4_^2−^ after ion exchange were optimized via DFT calculations. The calculations were performed at B3LYP-GD3BJ/SDD(Pd)/Iefpcm(water) and B3LYP-GD3BJ/6-31 + G × (other at-oms)/Iefpcm(water) using the Gaussian16 soft-ware package (Gaussian, Inc., New Haven, CT, USA). These levels are used to model the electronic structure and energetics of a system in the presence of water, with different basis sets being used for different types of atoms. The detailed frequency calculations of the optimized geometries are shown in Text S1. The molecular properties of the ion exchange products, including electrostatic potential (ESP) [46], molecular polarity index (MPI) [47], and interaction region indicator (IRI), were obtained by using the Multiwfn 3.8 (dev) program package (Tian Lu High-Technologies Corporation, Beijing, China) [48].

#### 2.3.1. Analysis of Ion Exchange Products

The analysis of the microemulsion system extraction products using IR and NMR showed that the extraction mechanism occurred via ion exchange between the imidazolium cation and metal complex anion. We utilized ESP analysis to further infer properties of target molecules and to determine the ion exchange interaction region [49]. The analysis was based on the molecular system formula of the ESP function.

(7) $$V_{\mathrm{tot}}\left(r\right){=V}_{\mathrm{nu}}\left(r\right){+V}_{\mathrm{ele}}\left(r\right)=\underset{A}{\sum }\frac{Z_{A}}{\left|{r-R}_{A}\right|}-\int \frac{{\rho (r}^{\prime })}{\left|{r-r}^{\prime }\right|}{\mathrm{dr}}^{\prime }$$

*Z* is the number of charges and *R* is the atomic coordinate. The static potential at point *r* was positive due to the dominance of atomic nuclear positive charge, but it was negative due to the influence of electrons. The ESP was defined as the interaction between a unit of positive charge placed at a particular point and the molecule at that point. ESP analysis was carried out using Gauss View 6.0 software (Gaussian, Inc., New Haven County, CT, USA), which generated a van der Waals surface color map that indicated the positive and negative ESP values.

**Figure 14:**
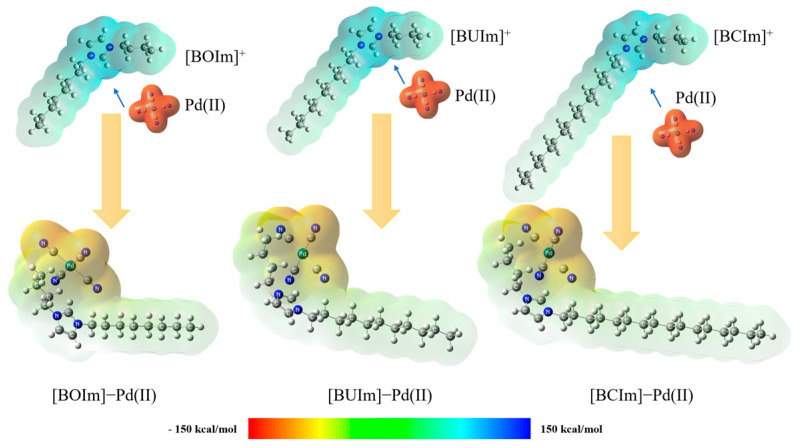
The color maps of the ESP distributions of [BOIm]^+^, [BUIm]^+^, [BCIm]^+^, and Pd(CN)_4_^2−^ and the ESP color map of the products after ion exchange.

Due to limited computational and hardware resources, we established a 1:1 binding model for Pd (II) and three different imidazolium cations to investigate the ion exchange mechanism system. The binding principles were similar to those of the 1:2 binding model. The ESP color map in Figure 14 indicates that the highest ESP positions for [BOIm]^+^, [BUIm]^+^, and [BCIm]^+^ were located near the imidazolium ring. Therefore, we inferred that the extraction active sites of [BOIm]^+^, [BUIm]^+^, and [BCIm]^+^ for Pd(CN)_4_^2−^ were located near the imidazolium ring. The calculations showed that the highest ESP of [BOIm]^+^, [BUIm]^+^, and [BCIm]^+^ were 123.89 kcal/mol, 123.92 kcal/mol, and 123.80 kcal/mol, respectively. This indicates that the electrostatic interaction strengths between the three extractants and Pd (II) were similar.

**Figure 15:**
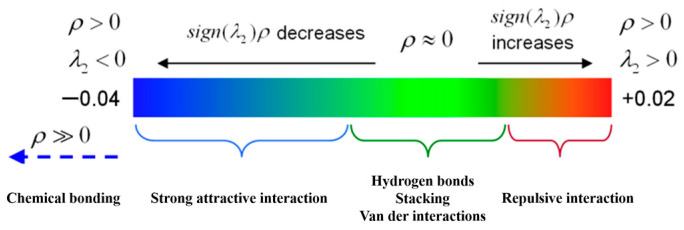
The strength and type of weak interactions.

To further analyze the bonds and weak interactions in the ion exchange products, we used an *IRI* method, as defined in Equation (8). The sign (λ_2_) ρ function was projected onto the *IRI* contour surface with different colors to differentiate the strength and characteristics of interactions in different regions. The *IRI* contour surface map was visualized using VMD (version 1.9.4a53) software (Theoretical and Computational Biophysics Group, Urbana, IL, USA). The blue region in the map indicates strong attractive interactions, with high ρ(r) values and small sign (λ_2_) *ρ* values. The green region had a value of ρ (*r*) ≈ 0, indicating weak interactions such as van der Waals forces and unconventional hydrogen bonding [50]. The red region had relatively large ρ (*r*) and sign (λ_2_) ρ values, indicating repulsive interactions. The RDG vs. sign (λ_2_) ρ scatter plots were visualized using gnuplot (version 5.4 patch-level 6) software (Figure 15).

(8) $$\mathrm{IRI}\left(r\right)=\frac{\left|\rho (r)\right|}{\left[\rho (r)\right]{\;}^{a}}$$

ρ is the electron density.

**Figure 16:**
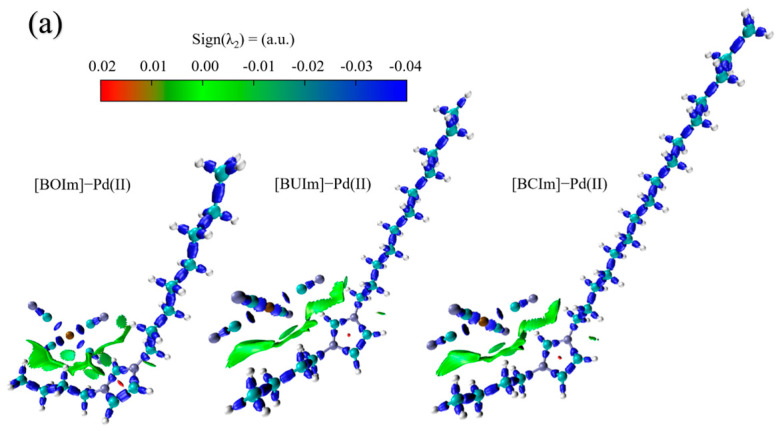

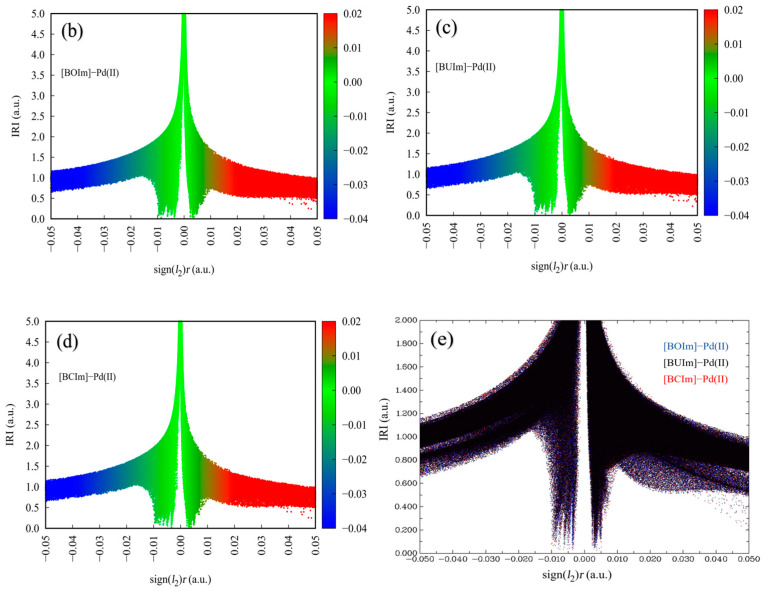
The IRI values for the three ion exchange product models (**a**), scatter plots of RDG vs. sign (λ_2_) for [BOIm][Pd(CN)_4_]^−^ (**b**), [BUIm][Pd(CN)_4_]^−^ (**c**), and [BCIm][Pd(CN)_4_]^−^ (**d**), and the enlarged comparison plots of the RDG vs. sign (λ_2_) ρ scatter plot data (**e**).

The IRI contour surface map of the [BOIm][Pd(CN)_4_]^−^, [BUIm][Pd(CN)_4_]^−^, and [BCIm][Pd(CN)_4_]^−^ models were shown in Figure 16a. The blue regions represent the original chemical bonds in the extracted product molecules. The red regions indicate steric hindrance within the imidazolium ring and the green regions indicate attractive interactions such as hydrogen bonding and van der Waals forces. Figure 16b–d shows the RDG vs. sign (λ_2_) ρ scatter plots of the [BOIm][Pd(CN)_4_]^−^, [BUIm][Pd(CN)_4_]^−^, and [BCIm][Pd(CN)_4_]^−^ models, as well as enlarged comparison plots of the three models. Multiple peaks can be observed in the range of −0.04 to 0.03 a.u. for sign (λ_2_) ρ, corresponding to weak interactions between the extractants and Pd (II). The extraction mainly occurred via electrostatic adsorption and weak interactions such as unconventional hydrogen bonding. Figure 16e shows enlarged comparison plots of the RDG vs. sign (λ_2_) ρ scatter plot data for the three ion exchange product models. The number and intensity of peaks in the weak interaction region were not significantly different, indicating that the extraction sites of [BOIm]^+^, [BUIm]^+^, and [BCIm]^+^ for Pd(CN)_4_^2−^ had similar attractive forces due to weak interactions.

**Figure 17:**
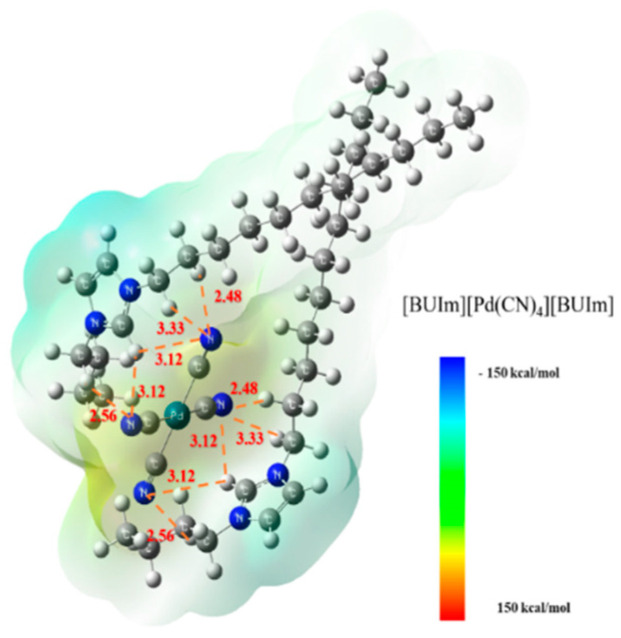
The color map of the ESP distributions of [BUIm][Pd(CN)_4_][BUIm].

Based on the actual experimental results, the main binding mode between the imidazolium cation and metal complex anions was 2:1. Taking the extracted ion exchange product [BUIm][Pd(CN)_4_][BUIm] as an example, as shown in Figure 17, the optimized product configuration was highly symmetrical. The cyanide groups of Pd (II) ions formed multiple hydrogen bonds with hydrogen atoms near the imidazolium ring. This was because the positively charged imidazolium group attracted the binding electrons of adjacent alkyl C–H bonds. This caused the H atoms of methylene groups to become positively charged, thus enabling the methylene H atom to act as a proton donor and form hydrogen bonds with the cyanide group’s N atom. The optimized results were consistent with the IR and NMR spectra, indicating that this structure enhanced the ion binding effect and promoted extraction. The surface ESP of the extracted compound became more neutral after binding, which enabled the metal complexes to be separated from the water after being extracted with the microemulsion system. (The surface analysis results of ESP values are as follows: the maximum value of the [BUIm]^+^ molecular surface was 123.92 kcal/mol, the minimum value of the [Pd(CN)_4_]^2−^ molecular surface was −187.76 kcal/mol, while the maximum and minimum values of the [BUIm][Pd(CN)_4_][BUIm] molecular surface were 52.19 and −68.16 kcal/mol, respectively.)

#### 2.3.2. Analysis of Extractant in the Organic Phase

According to the extraction experiment results, [BUIm]^+^ exhibited better extraction efficiency for metal complexes in water compared with [BOIm]^+^ and [BCIm]^+^. Therefore, other factors may ultimately lead to differences in extraction efficiency when the three extractants have similar interactions with Pd (II). Based on previous studies [51,52,53], it is speculated that during extraction, the organic phase (n-octane) contains extractant–alcohol–water complexes, which form a “double layer structure” at the oil–water interface to form a microemulsion system. Then, metal complexes are extracted into water. After extraction, appropriate electrolytes are used to destroy the double layer structure at the oil–water interface, which increases the specific gravity of the aqueous phase, thereby achieving phase separation. The stability of the microemulsion extraction system depends on the hydrophilic–lipophilic balance of the extractant–alcohol–water complexes. Only when the hydrophilic–lipophilic balance of the complexes reaches equilibrium can the microemulsion system exist stably and achieve the highest microemulsion stability area to achieve the best extraction effect. In this microemulsion system, the imidazolium salt simultaneously acted as a surfactant and extractant, while 1-pentanol was a co-surfactant and n-heptane was the organic phase.

Three binding models were established and analyzed for the complexes formed by [BOIm]^+^, [BUIm]^+,^ and [BCIm]^+^ dissolved in n-octane/1-pentanol. The structures were optimized, and frequency calculations were performed using DFT. The MPI was obtained through molecular surface analysis based on the calculated molecular ESP data and used to compare the molecular polarity of similar molecules [46]. The MPI was calculated using Equation (9).

(9) $$\mathrm{MPI}=\frac{1}{A}\iint \left|V\left(r\right)\right|\mathrm{dS}$$

A represents the area of the van der Waals surface of the substance molecule, and the integral is numerically evaluated through the molecular van der Waals surface (*S*); *V*(*r*) represents the ESP value at point r in space.

**Figure 18:**
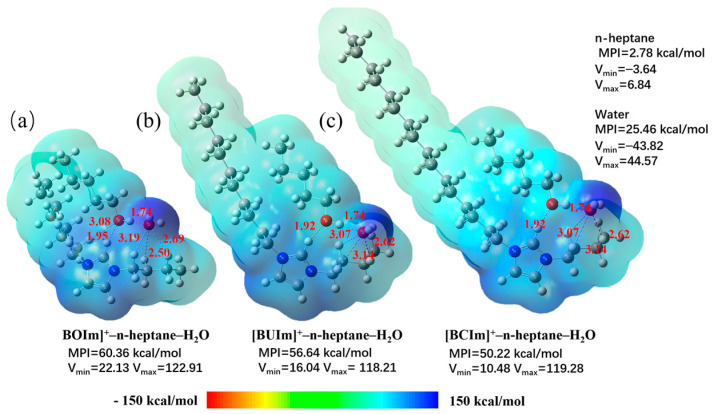
The color maps of the ESP distributions of [BOIm]^+^—n-heptane-H_2_O (**a**), [BUIm]^+^—n-heptane-H_2_O (**b**), and [BCIm]^+^—n-heptane-H_2_O (**c**).

As shown in Figure 18, the MPI values of these extractant–alcohol–water complexes were all very high because the surface ESP of all complexes was dominated by the extractant active site cation. This indicatesd that the polar portions of these molecules all have very high electrostatic potentials and they are highly hydrophilic. However, after extracting metal complexes, the MPI values decreased significantly, indicating that the polarity decreased, and the complexes were more soluble in the oil phase (the MPI values are as follows: Pd(CN)_4_^2−^ before extraction was 174.26 kcal/mol, [BUIm]^+^ was 61.32 kcal/mol, [BUIm][Pd(CN)_4_][BUIm] was 17.59 kcal/mol, and n-heptane was 2.78 kcal/mol). The order of MPI values for the three binding models is [BOIm]^+^-n-heptane-H_2_O > [BUIm]^+^-n-heptane- H_2_O > [BCIm]^+^-n-heptane- H_2_O, indicating that the microemulsion extraction system formed by the three binding models had the highest distribution ratio in the water phase before extraction. [BOIm]^+^-n-heptane-H_2_O had the lowest distribution ratio in the oil phase after extraction, while [BCIm]^+^-n-heptane-H_2_O was the opposite, and [BUIm]^+^-n-heptane-H_2_O was between these two systems [54]. Observing the optimized structures of the three models shows that due to steric hindrance, [BOIm]^+^-n-heptane-H_2_O formed the shortest and most stable average hydrogen bond length. 

Therefore, it is speculated that mutual weak interactions between the extractant–alcohol–water complexes were stronger, which reduced the solubility of alcohol in the microemulsion system, reduced the amount of water added, and decreased the mobility of the double-layer membrane at the oil–water interface of the microemulsion system, thus increasing its rigidity. Finally, the spontaneous decrease in the microemulsion system decreased the microemulsion stability area and extraction rate. Based on the above analysis, it can be concluded that of the three models, the microemulsion extraction systems of [BUIm]^+^-n-heptane-H_2_O and [BCIm]^+^-n-heptane-H_2_O were more stable. Since the hydrophilicity of [BUIm]^+^-n-heptane-H_2_O was better than that of [BCIm]^+^-n-heptane-H_2_O, [BUIm]^+^-n-heptane-H_2_O had the best extraction performance for negatively charged metal complexes in water. This was consistent with the extraction experiment results, so [BUIm]^+^ was determined to be the optimal extractant.

## 3. Materials and Methods

### 3.1. Instruments and Reagents

Nuclear magnetic resonance (NMR) spectra of the as-synthesized imidazolium-based ionic liquids and the extracted complexes were measured with an NMR spectrometer (Avance 600, Bruker, Karlsruhe, Germany). The synthesized and extracted species were characterized using a Fourier-transform infrared spectrometer (NicoletiS10, Thermo Fisher Scientific, Waltham, MA, USA). UV spectra of [BUIm]Br, Pd(CN)_4_^2−^, and [BUIm]-Pd (II) were recorded using a UV–Vis spectrophotometer (UV-2550, Shimadzu Corporation, Kyoto, Japan). The chemical composition of the synthesized and extracted species was analyzed using a mass spectrometer (1100HPLC/TOF, Agilent Instrument Corporation, Santa Clara, CA, USA). The concentrations of Pd(CN)_4_^2−^, Fe(CN)_6_^3−^, and Co(CN)_6_^3−^ were determined through atomic absorption spectrometry (Z-3000, Hitachi High-Technologies Corporation, Tokyo, Japan). Extraction experiments were performed in a thermostatic shaker (ZQTY-70V, ZhiChu Instrument Corporation, Shanghai, China).

N-pentanol was purchased from Sinopharm Chemical Reagent Co., Ltd. (Beijing, China), n-heptane was purchased from Tianjin Windboat Chemical Reagent Technology Co., Ltd. (Tianjin, China), 1-butylimidazole and 1-undecane bromide were purchased from Titan Technology Co., Ltd. (Beijing, China). All reagents were analytical reagents and were used without further purification.

### 3.2. Synthesis of Ionic Liquids

Three imidazolium-based ionic liquids, viz., [BUIm]Br, [BOIm]Br, and [BCIm]Br, were synthesized. Of these, [BUIm]Br is a new ionic liquid. The synthesis of [BUIm]Br was as follows: 1-butylimidazole (0.05 mol, 6.21 g) and 1-bromoundecane (0.05 mol, 11.76 g) were added to a 50 mL round-bottom flask and slowly brought to 80 °C with continuous stirring for 12 h. The coarse products were then washed several times with ether (3 × 20 mL) and dried under vacuum for 12 h to obtain [BUIm]Br.

[BUIm]Br was characterized using Fourier-transform infrared (FT-IR), nuclear magnetic resonance (NMR), and mass spectrometry (MS). FTIR (cm^−1^): 3033(=C–H stretching vibration of imidazolium ring), 3071 (C–H stretching vibration near imidazolium ring), 1563 and 1632 (C=N and C=C stretching vibration of imidazolium ring). ^1^H NMR (600 MHz, CDCl_3_) δ 10.38 (d, J = 1.7 Hz, 1H, –CH), 7.47 (t, J = 1.8 Hz, 1H, –CH), 7.42 (d, J = 1.8 Hz, 1H, –CH), 4.32 (dt, J = 16.4, 7.4 Hz, 4H, –CH_2_), 2.18 (s, 2H, –CH_2_), 1.94–1.81 (m, 4H, –CH_2_), 1.37–1.17 (m, 18H, –CH_2_), 0.92 (t, J = 7.4 Hz, 3H, –CH_3_), 0.83 (t, J = 7.0 Hz, 3H, –CH_3_). ^13^C NMR (150 MHz, CDCl_3_) δ 137.00, 122.01, 121.82, 50.00, 49.71, 32.04, 31.70, 30.18, 29.37, 29.21, 28.84, 26.10, 22.48, 19.32, 13.91, 13.29. HR-MS(ESI^+^): [M]^+^ calcd: (C_18_H_35_N_2_^+^)279.2795; found, 279.2797. The IR, NMR, and MS spectra of [BUIm]Br are shown in Figures S1–S3. 

Two other imidazolium-based ionic liquids, [BOIm]Br and [BCIm]Br, were synthesized with reference to the literature [55,56]. The synthetic procedures for the three ionic liquids are shown in Table S1, and IR, NMR, and MS spectra of [BOIm]Br and [BCIm]Br are shown in Figures S4–S9.

### 3.3. Preparation of ILs/n-Heptane/n-Pentanol/NaCl Microemulsion

Different masses of [BUIm]Br, [BCIm]Br, or [BOIm]Br were added to a mixture of n-heptane and n-pentanol (30 vol%). Sodium chloride solution was slowly added until the water phase appeared, and the samples were allowed to equilibrate for several hours. The organic phase was completely separated from the water phase, and the transparent organic phase was the n-heptane/n-pentanol/sodium chloride microemulsion system.

### 3.4. Extraction Process

Extraction experiments were performed by mixing 10 mL microemulsion phase with 10 mL water phase at a phase ratio (*R_A/O_*) of 1:1 in a 50 mL centrifuge tube. Then, the tube was placed in a thermostatic shaker for 10 min (25 °C, 240 rpm). After the end of the reaction, it was separated. Atomic absorption spectroscopy(AAS) was used to determine the concentration of metal ions in the water phase before and after extraction [33].

The extraction efficiency (%), distribution ratio (*D*), and separation faction (*K*) were calculated using Equations (10)–(13), respectively.

(10) $$E\%=\frac{c_{in}-c_{eq}}{c_{in}}\times 100$$

(11) $$D=\frac{c_{\mathrm{or}}}{c_{\mathrm{eq}}}\times R_{A/O}\;\;$$

(12) $$K=\frac{D_{Pd}}{D_{M}}\;\;$$

where $C_{in}$ and $C_{eq}$ indicate the original and immediate concentrations of Pd(CN)_4_^2−^ in the water phase, respectively.$\;C_{or}$ indicates the concentration of Pd(CN)_4_^2−^ in the organic phase. *R_A/O_* represents the volume ratio of the aqueous phase to the organic phase.

### 3.5. Recovery of Palladium from the Organic Phase

The reverse extractant solution was mixed at a phase ratio of 5:1 to strip palladium. The effects of potassium bromide, potassium iodide, sodium hydroxide, and potassium thiocyanate on the stripping of palladium were investigated. Then, the effects of the concentration of the stripping agent and the oscillation time on the stripping effect were explored.

The stripping efficiency (*S*%) was calculated as follows:

(13) $$S\%=\frac{c_{\mathrm{or}}-c_{\mathrm{eq}}}{c_{\mathrm{or}}}\times 100$$

## 4. Conclusions

A new imidazolium ionic liquid [BUIm]Br was synthesized, and a [BUIm]Br/heptane/pentanol/NaCl microemulsion system was made to extract Pd(CN)_4_^2−^. ^1^H NMR, FT-IR, and UV analysis confirmed that the Pd (II) extraction mechanism occurred via anion exchange. The effects of [BUIm]Br concentration, extraction time, and pH were investigated. The results showed that the extraction was an exothermic reaction, as the extraction rate decreased when the temperature increased. DFT calculations were used to compare three imidazolium ionic liquid extractant models, ESP, MPI, and IRI data, to investigate the factors affecting the extraction efficiency of the microemulsion system. Extraction relied mainly on electrostatic adsorption and unconventional hydrogen bonding, in addition to the double-layer structure of the oil–water interface, which also played a key role in extraction. Under optimal conditions, the [BUIm]Br/heptane/pentanol/NaCl system selectively recovered Pd (II) from an alkaline solution containing Co (III) and Fe (III). After five extraction cycles, the extraction rate remained higher than 90% when using 0.5 mol·L^−1^ KSCN. The [BUIm]Br/heptane/pentanol/NaCl microemulsion system completely separated Pd (II) from an alkaline cyanide solution. 

## Figures and Tables

**Table 1:** The stripping rates of different stripping reagents for Pd (II).

Reverse Extractant	Extraction Rate (%)	Stripping Extraction (%)
KBr	99.7	76.1
KI	99.8	91.6
NaOH	99.8	7.9
KSCN	99.8	99.5

**Table 2:** Separation of Pd(CN)_4_^2−^ from Fe(CN)_6_^3−^ and Co(CN)_6_^3−^.

E%		S%		R%
		1 mol·L^−1^ KCl	0.5 mol·L^−1^ KSCN	
Pd(CN)_4_^2−^	99.2	2.9	96.1	95.3
Fe(CN)_6_^3−^	98.7	97.8	-	96.5
Co(CN)_6_^3−^	98.5	98.0	-	96.5

**Table 3:** Reusability of [BUIm]Br in single Pd (II) solutions.

Time	Extraction Rate (%)	Recovery Rate (%)
1	99.2	96.5
2	98.5	94.8
3	97.3	93.2
4	94.8	91.6
5	94.8	90.7

**Table 4:** Vibration bands associated with [BUIm]Br and [BUIm]Br-Pd (II).

[BUIm]Br Bands (cm^−1^)	[BUIm]Br-Pd (II) Bands (cm^−1^)	Vibration
3424	3425	stretching vibration of –OH
3133	3140	=C–H stretching of imidazolium ring
3071	3090	C–H stretching near imidazolium ring
2963	2959	C–H stretching of CH_3_
2854	2856	C–H stretching of CH_2_
-	2126	C≡N stretching vibration of Pd(CN)_4_^2−^
1632	1642	C=C stretching of imidazolium ring
1563	1565	C=N stretching of imidazolium ring
1465	1466	C–H stretching of CH_2_

**Table 5:** Thermodynamic parameters of Pd (II) extraction by [BUIm]Br/heptane/pentyl alcohol/NaCl microemulsion system.

Temperature (K)	ΔG (KJ mol^−1^)	ΔH (KJ mol^−1^)	ΔS (J mol^−1^ K^−1^)
298	−19.10	−38.37	−64.82
303	−18.71		
308	−18.32		
313	−18.17		
318	−17.75		

## Data Availability

Not applicable.

## Supplementary Materials

The supporting information can be downloaded at: https://www.mdpi.com/article/10.3390/ijms241310709/s1.
