# Supplementary material for: Separation of Palladium from Alkaline Cyanide Solutions through Microemulsion Extraction Using Imidazolium Ionic Liquids
Source: Int J Mol Sci. 2023 Jun 27;24(13):10709. doi: 10.3390/ijms241310709 (PMC10341638; doi:10.3390/ijms241310709)
Supplement: Supplementary file 1 [file ijms-24-10709-s001.zip › ijms-2414030-supplementary.pdf]

Supplementary Materials

Separation of palladium from alkaline cyanide solutions by microemulsion extraction using imidazolium ionic liquids

Table S1. Synthesis and characterization of extractant.

Sample	Synthetic scheme	Characterization
1-butyl-3- undecyl imidazolium bromide ([BUIm]Br)	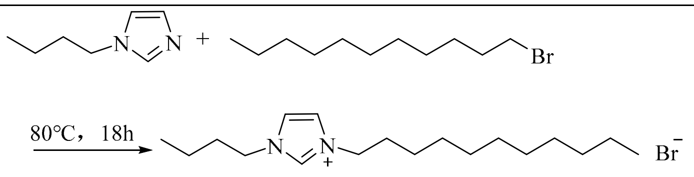	IR: Figure S1<br>NMR: Figure S2<br>MS: Figure S3
1-butyl-3-octyl imidazolium bromide ([BOIm]Br	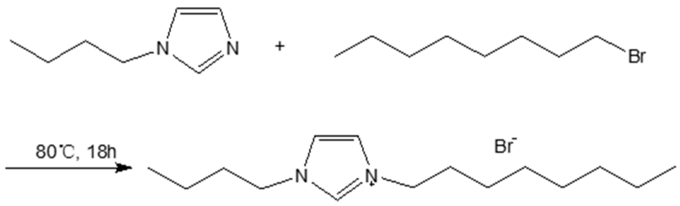	IR: Figure S4<br>NMR: Figure S5<br>MS: Figure S6
1-butyl-3-hexadecyl imidazolium bromide ([BCIm]Br)	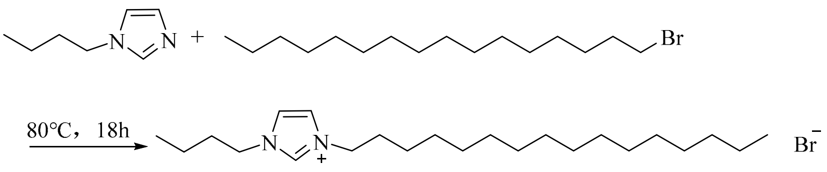	IR: Figure S7<br>NMR: Figure S8<br>MS: Figure S9

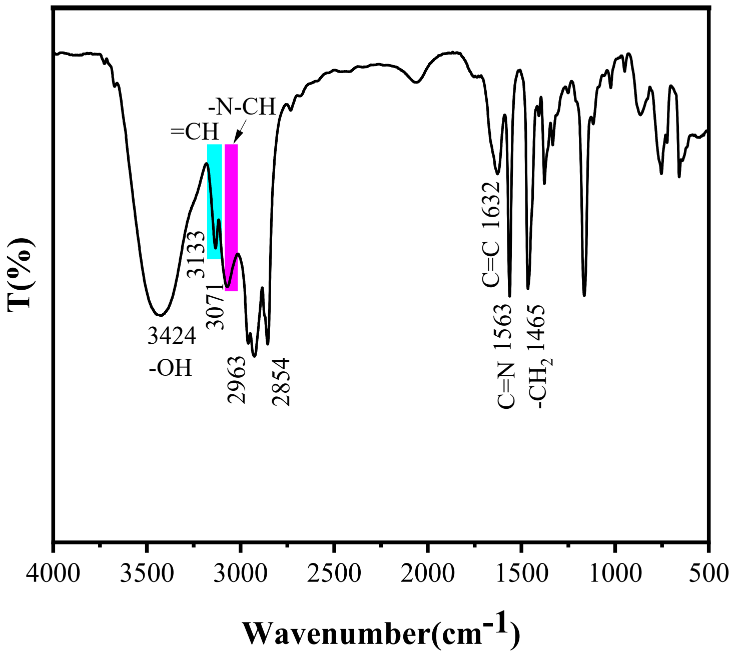

Figure S1. IR spectrum of [BUIm]Br

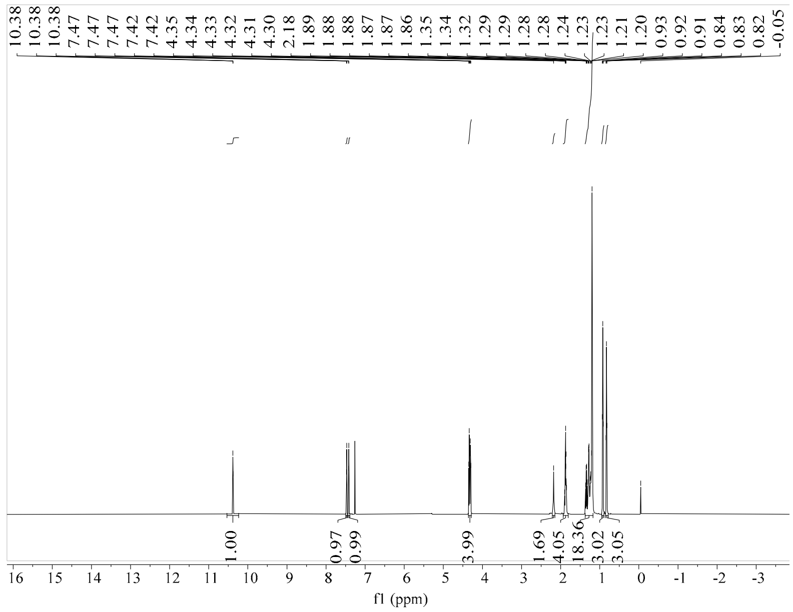

^{1}H NMR (600 MHz, $CDCl_3$) δ 10.38 (d, J = 1.7 Hz, 1H, CH), 7.47 (t, J = 1.8 Hz, 1H, CH), 7.42 (d, J = 1.8 Hz, 1H, CH), 4.32 (dt, J = 16.4, 7.4 Hz, 4H, -CH_2), 2.18 (s, 2H-CH_2), 1.94 – 1.81 (m, 4H-CH_2), 1.37 – 1.17 (m, 18H, -CH_2), 0.92 (t, J = 7.4 Hz, 3H-CH_3), 0.83 (t, J = 7.0 Hz, 3H, -CH_3).

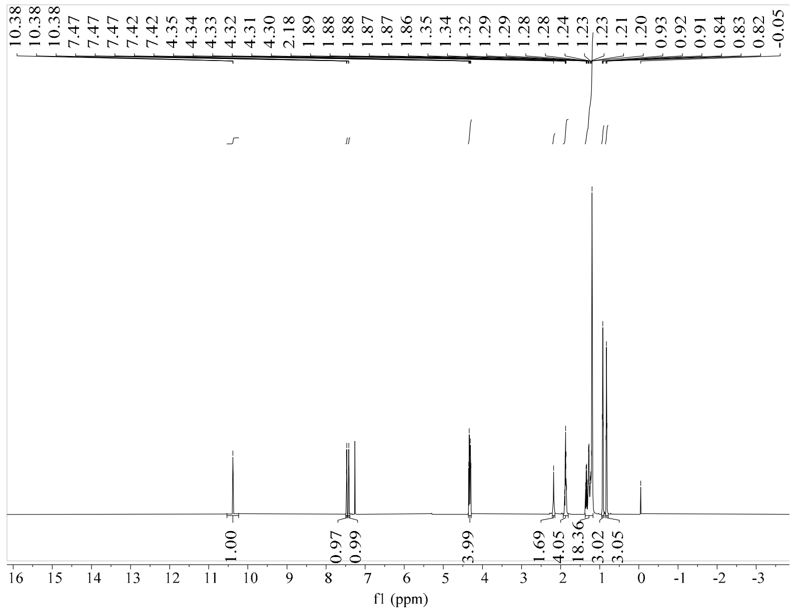

^{13}C NMR (150 MHz, $CDCl_3$) δ 137.00, 122.01, 121.82, 50.00, 49.71, 32.04, 31.70, 30.18, 29.37, 29.21, 28.84, 26.10, 22.48, 19.32, 13.91, 13.29.

Figure S2. NMR spectra of [BUIm]Br

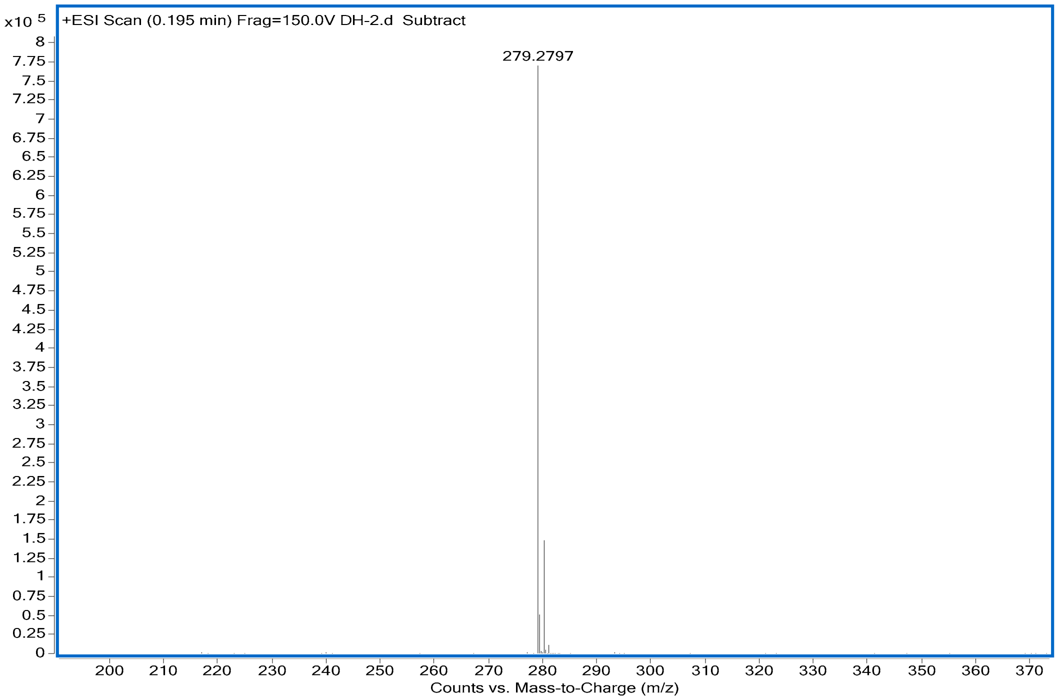

Figure S3. HR-MS(ESI$^+$): [M]+ calcd: ($C_{18}H_{35}N_2^+$)279.2795; found, 279.2797.

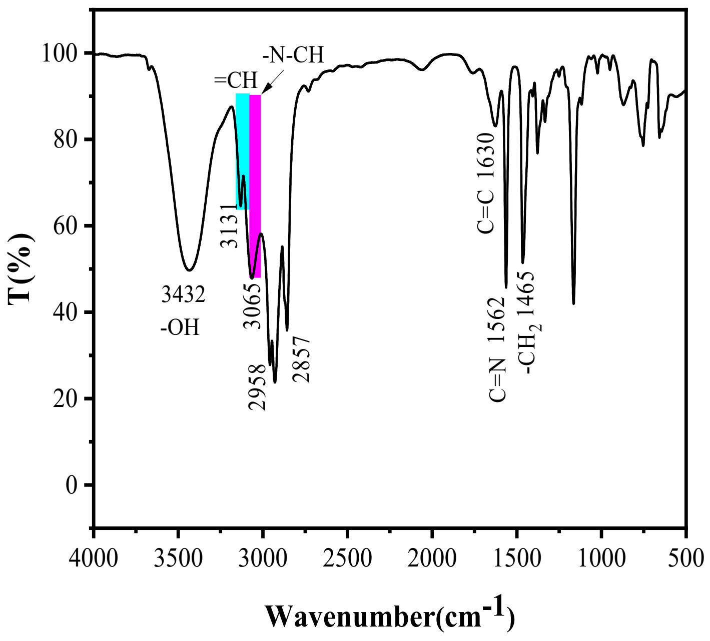

Figure S4. IR spectrum of [BOIm]Br.

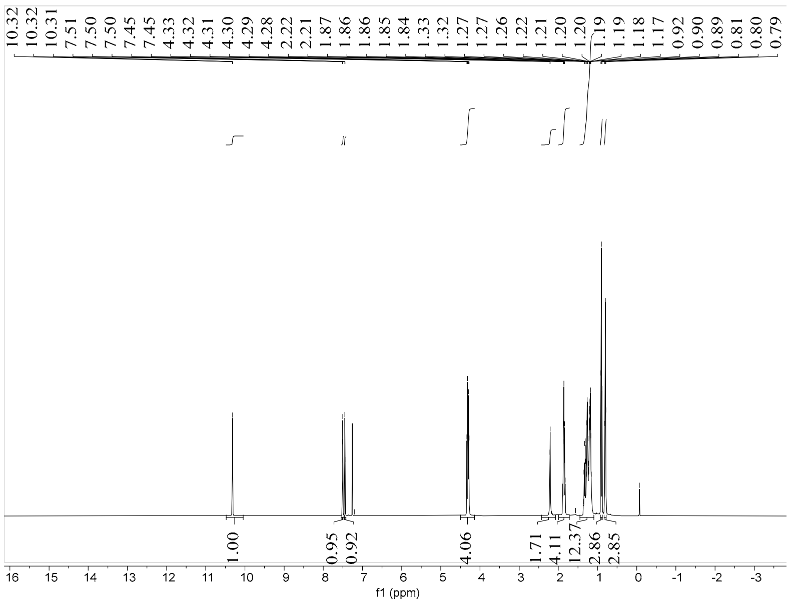

^{1}H NMR (500 MHz, $CDCl_3$) δ 10.31 (d, J = 1.7 Hz, 1H, CH), 7.50 (t, J = 1.8 Hz, 1H, CH), 7.45 (d, J = 1.8 Hz, 1H, CH), 4.31 (dt, J = 12.7, 7.4 Hz, 4H, $-CH_2$), 2.21 (d, J = 3.0 Hz, 2H, $-CH_2$), 1.99 – 1.72 (m, 4H $-CH_2$), 1.45 – 1.10 (m, 12H, $-CH_2$), 0.90 (t, J = 7.4 Hz, 3H, $-CH_3$), 0.80 (t, J = 6.8 Hz, 3H, $-CH_3$)

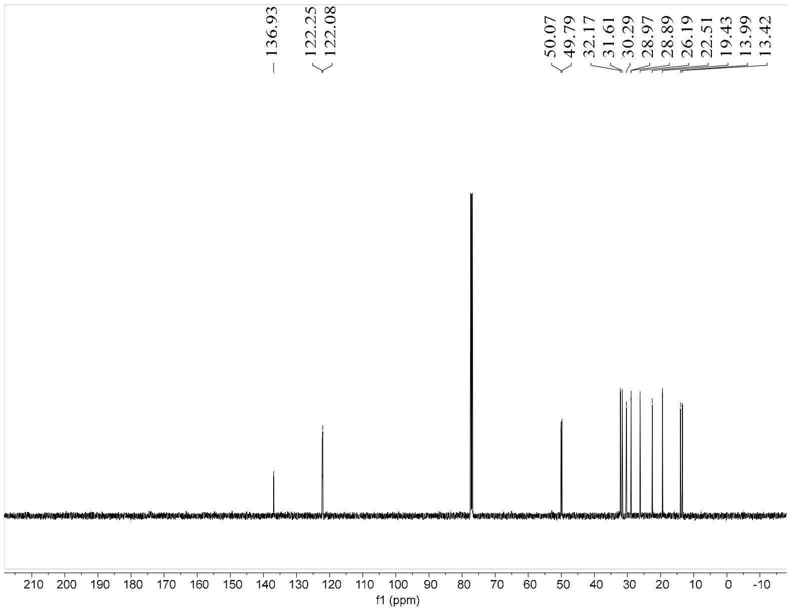

^{13}C NMR (125 MHz, $CDCl_3$) δ 136.93, 122.25, 122.08, 50.07, 49.79, 32.17, 31.61, 30.29, 28.97, 28.89, 26.19, 22.51, 19.43, 13.99, 13.42.

Figure S5. NMR spectra of [BOIm]Br.

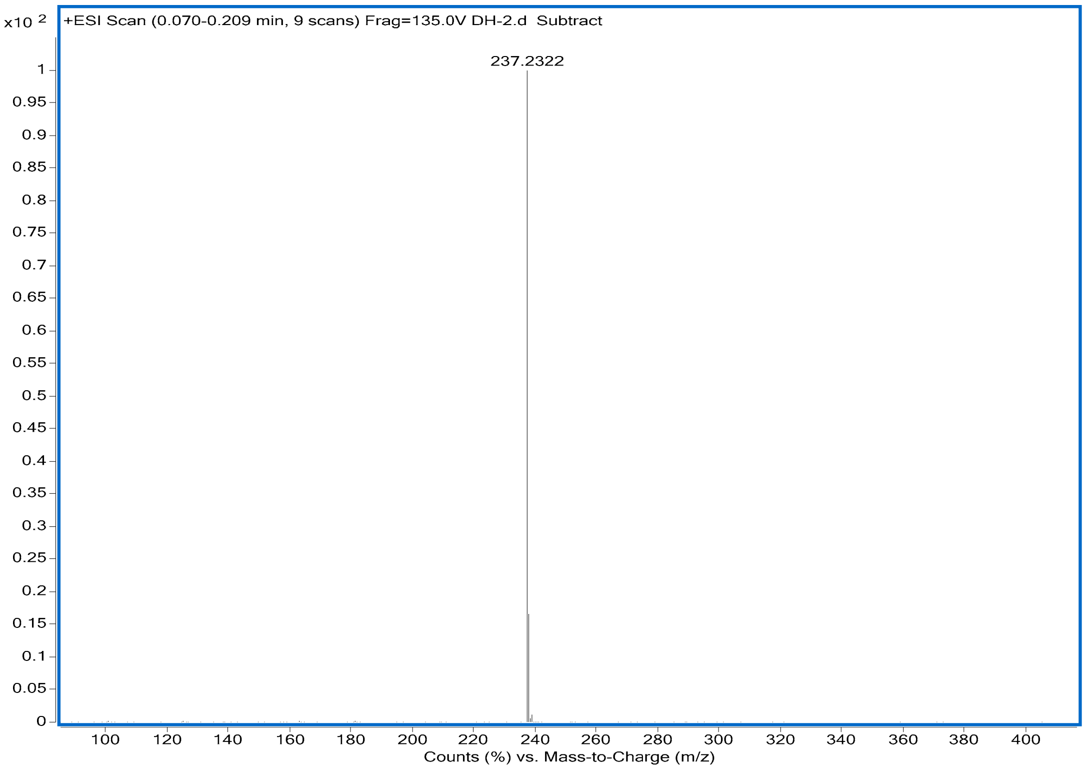

Figure S6. HR-MS(ESI$^+$): [M]+ calcd: ($C_{15}H_{29}N_2^+$)237.2325; found, 237.2322.

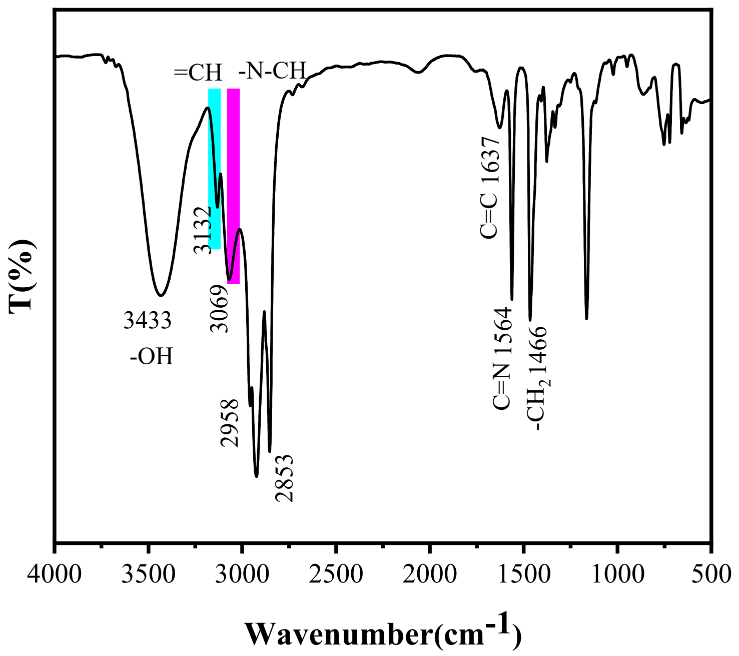

Figure S7. IR spectrum of [BCIm]Br

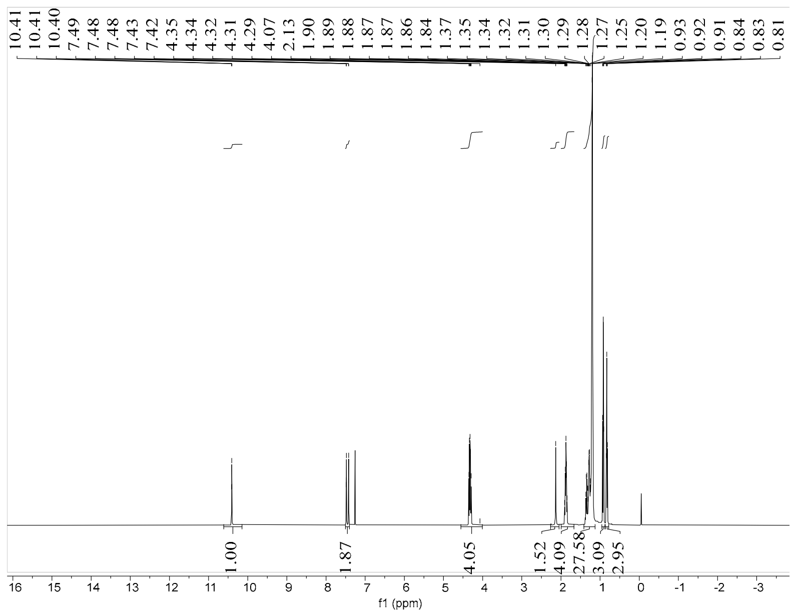

^{1}H NMR (500 MHz, $CDCl_3$) δ 10.41 (d, *J* = 1.8 Hz, 1H, CH), 7.51 – 7.40 (m, 2H, CH), 4.32 (dt, *J* = 14.5, 7.5 Hz, 4H, CH_2), 2.13 (s, 2H, CH_2), 2.00 – 1.67 (m, 4H, CH_2), 1.41 – 1.13 (m, 28H, CH_2), 0.92 (t, *J* = 7.4 Hz, 3H, CH_3), 0.83 (t, *J* = 6.9 Hz, 3H, CH_3).

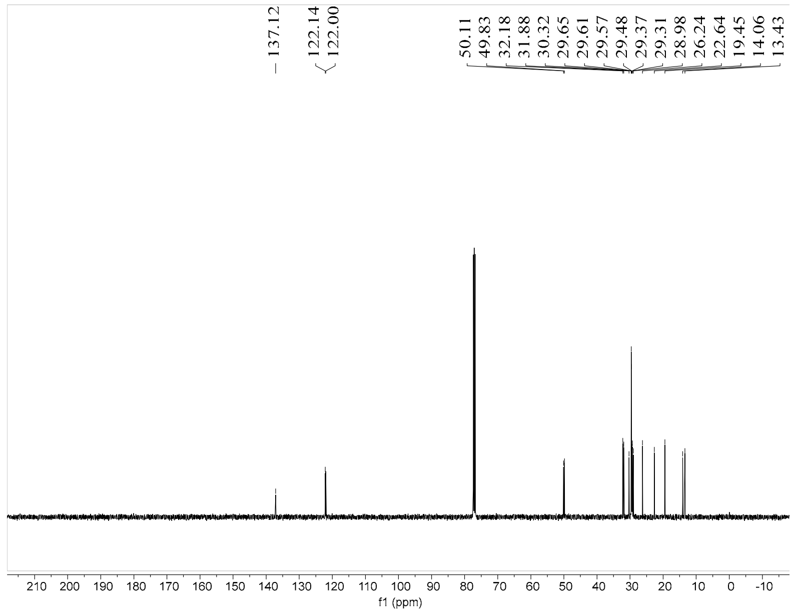

^{13}C NMR (125 MHz, $CDCl_3$) δ 137.12, 122.14, 122.00, 50.11, 49.83, 32.18, 31.88, 30.32, 29.65, 29.61, 29.57, 29.48, 29.37, 29.31, 28.98, 26.24, 22.64, 19.45, 14.06, 13.43.

Figure S8. NMR spectra of [BCIm]Br

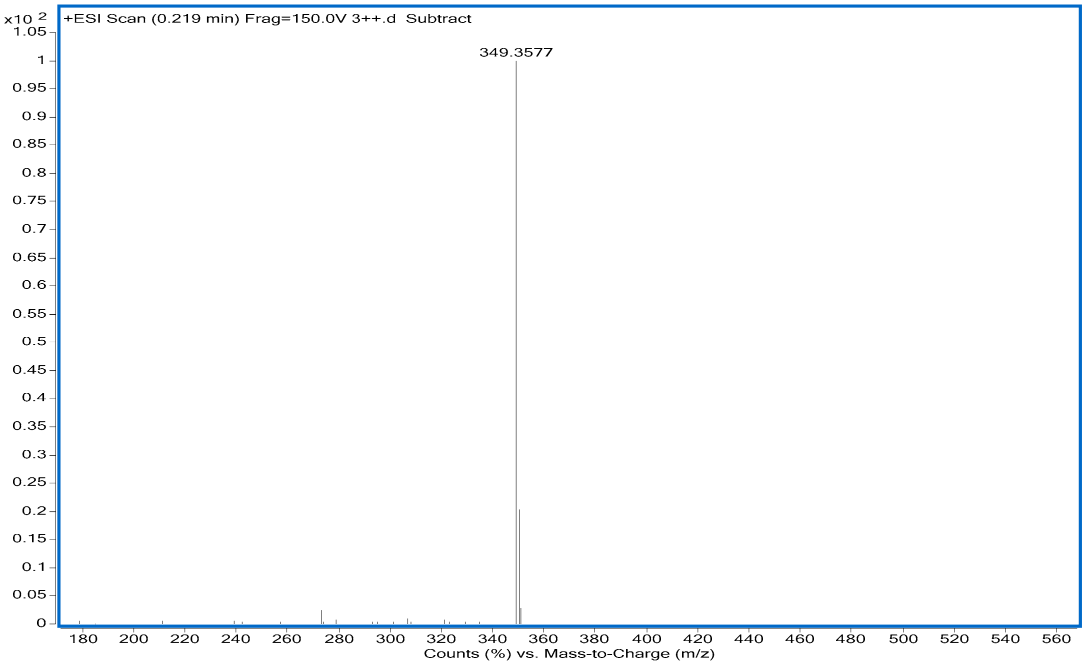

Figure S9. HR-MS(ESI$^+$): [M]+ calcd: ($C_{23}H_{45}N_2^+$)349.3577; found, 349.3577.

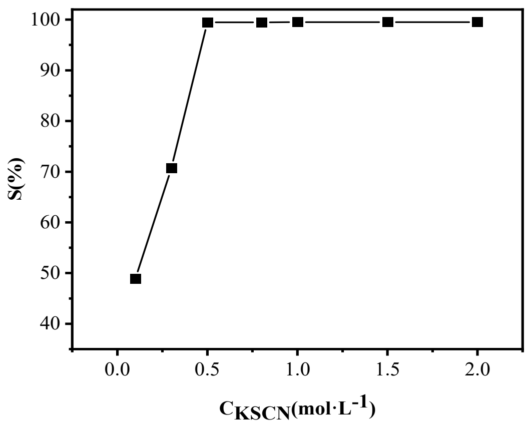

Figure S10. Influence of C_{KSCN}

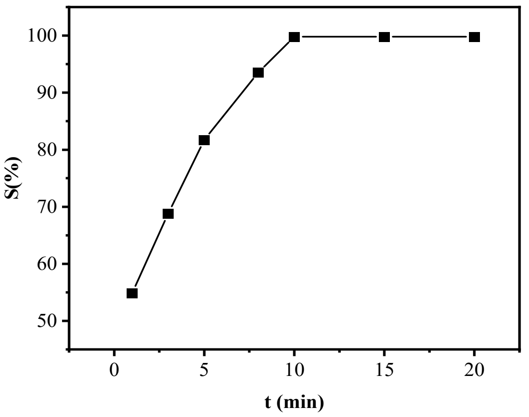

Figure S11. Influence of stripping extraction time

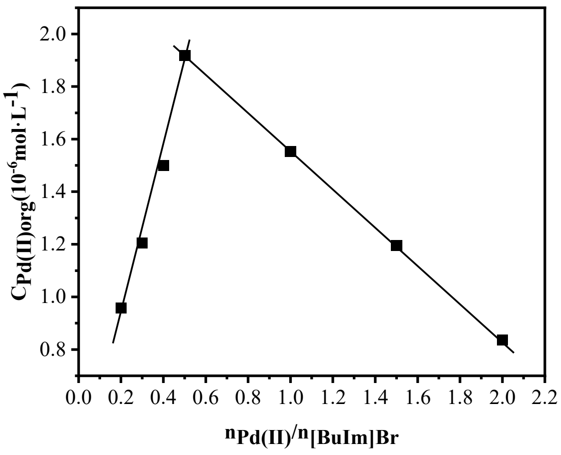

Figure S12. Continuous variation method of precipitation reaction between Pd(II) and [BUIm]Br

Text S1 Optimized structure and analysis

$Pd(CN)_4^{2-}$

-2 1

Pd	-0.00005189	0.00024577	0.00000962
C	1.37705159	-1.46668471	0.00031028
N	2.17190400	-2.31510579	0.00112008
C	-1.37749565	1.46695746	0.00024128
N	-2.17308521	2.31469763	0.00099666
C	-1.46729063	-1.37675660	0.00023682
N	-2.31541811	-2.17192148	-0.00075140
C	1.46766164	1.37667438	-0.00041028
N	2.31700294	2.17055126	-0.00175267

Volume: 1207.42149 Bohr^3 (178.92141 Angstrom^3)

Estimated density according to mass and volume (M/V): 1.9535 g/cm^3

Minimal value: -187.76398 kcal/mol Maximal value: -166.11178 kcal/mol

Overall surface area: 662.01850 Bohr^2 (185.38406 Angstrom^2)

Positive surface area: 0.00000 Bohr^2 (0.00000 Angstrom^2)

Negative surface area: 662.01850 Bohr^2 (185.38406 Angstrom^2)

Overall average value: -0.27770858 a.u. (-174.26491 kcal/mol)

Positive average value: NaN a.u. (NaN kcal/mol)

Negative average value: -0.27770858 a.u. (-174.26491 kcal/mol)

Overall variance (sigma^2_tot): 0.00005726 a.u.^2 (22.54614 (kcal/mol)^2)

Positive variance: 0.00000000 a.u.^2 (0.00000 (kcal/mol)^2)

Negative variance: 0.00005726 a.u.^2 (22.54614 (kcal/mol)^2)

Balance of charges (nu): 0.00000000

Product of sigma^2_tot and nu: 0.00000000 a.u.^2 (0.00000 (kcal/mol)^2)

Internal charge separation (Pi): 0.00607867 a.u. (3.81442 kcal/mol)

Molecular polarity index (MPI): 7.55683477 eV (174.26491 kcal/mol)

Nonpolar surface area (|ESP| <= 10 kcal/mol): 0.00 Angstrom^2 (0.00 %)

Polar surface area (|ESP| > 10 kcal/mol): 185.38 Angstrom^2 (100.00 %)

[BOIm]$^{+}$

1 1

C	-2.33590794	-1.02018018	-0.80855550
N	-1.39693915	-1.64101225	-0.08936469
C	-1.92990616	-1.96705478	1.14433123
C	-3.21892521	-1.53260314	1.14923368
N	-3.45379902	-0.94719341	-0.08122365
C	-4.70300570	-0.28417316	-0.49185160
C	-4.81591310	1.12172294	0.09218131
C	-6.11460411	1.80704409	-0.33712363
C	-6.24625584	3.21589922	0.24099056
C	-0.00244904	-1.86282702	-0.50919114
C	0.92628705	-0.77351989	0.02124457
C	2.37663135	-1.01191779	-0.40047554
C	3.32367047	0.07257363	0.11598820
H	-2.21314084	-0.64392457	-1.80946228
H	-1.35467682	-2.48163251	1.89411491
H	-3.98419276	-1.59685335	1.90286154
H	-4.71236121	-0.26336420	-1.58104353
H	-5.52583235	-0.92010978	-0.16508296
H	-4.76823939	1.06555942	1.18467256
H	-3.95405260	1.71353394	-0.23327948
H	-6.15705281	1.85250823	-1.43124291
H	-6.96891972	1.19779919	-0.02126724
H	-7.17901303	3.68814407	-0.07698880
H	-6.23877672	3.19663008	1.33476695
H	-5.42099122	3.85569495	-0.08518480
H	0.28790040	-2.84866658	-0.14571067
H	0.00502077	-1.89599023	-1.59837839
H	0.58262332	0.19785406	-0.34881886

H	0.85693462	-0.73964772	1.11324847
H	2.70786568	-1.99061953	-0.03310892
H	2.43669841	-1.05752666	-1.49448473
H	2.98817969	1.05065341	-0.25068258
H	3.25862795	0.11891694	1.21018798
C	4.77927243	-0.15118515	-0.29613210
H	5.11389024	-1.12995231	0.07056339
H	4.84328357	-0.19905991	-1.39067444
C	5.72669258	0.93319475	0.21923078
H	5.66172734	0.98190120	1.31396567
H	5.39174688	1.91223259	-0.14731226
C	7.18462829	0.71396647	-0.18966420
H	7.51923876	-0.26412594	0.17691988
H	7.24947431	0.66573435	-1.28349908
C	8.12258708	1.80351247	0.33084056
H	9.15717988	1.62416310	0.02539675
H	7.83166638	2.78883562	-0.04683333
H	8.10354564	1.85203700	1.42419246

Volume: 2513.28489 Bohr^3 (372.43040 Angstrom^3)

Estimated density according to mass and volume (M/V): 1.0585 g/cm^3

Minimal value: 22.81997 kcal/mol Maximal value: 123.89007 kcal/mol

Overall surface area: 1282.52742 Bohr^2 (359.14426 Angstrom^2)

Positive surface area: 1282.52742 Bohr^2 (359.14426 Angstrom^2)

Negative surface area: 0.00000 Bohr^2 (0.00000 Angstrom^2)

Overall average value: 0.10823597 a.u. (67.91915 kcal/mol)

Positive average value: 0.10823597 a.u. (67.91915 kcal/mol)

Negative average value: NaN a.u. (NaN kcal/mol)

Overall variance (sigma^2_tot): 0.00174764 a.u.^2 (688.16657 (kcal/mol)^2)

Positive variance: 0.00174764 a.u.^2 (688.16657 (kcal/mol)^2)

Negative variance: 0.00000000 a.u.^2 (0.00000 (kcal/mol)^2)

Balance of charges (nu): 0.00000000

Product of sigma^2_tot and nu: 0.00000000 a.u.^2 (0.00000 (kcal/mol)^2)

Internal charge separation (Pi): 0.03664592 a.u. (22.99568 kcal/mol)

Molecular polarity index (MPI): 2.94525058 eV (67.91915 kcal/mol)

Nonpolar surface area (|ESP| <= 10 kcal/mol): 0.00 Angstrom^2 (0.00 %)

Polar surface area (|ESP| > 10 kcal/mol): 359.14 Angstrom^2 (100.00 %)

$[BUIm]^{+}$

1 1

C	-4.12790130	-0.95685650	-0.78411970
N	-3.22078564	-1.61533056	-0.05781865
C	-3.74954993	-1.85442711	1.19735785
C	-5.00376792	-1.32816684	1.20841383
N	-5.22222032	-0.77524066	-0.04000610
C	-6.43343737	-0.04849780	-0.45652919
C	-6.45597186	1.37770423	0.08731726
C	-7.71383957	2.12824926	-0.35478488
C	-7.75262646	3.56048065	0.17771619
C	-1.85916990	-1.96631810	-0.49611782
C	-0.82270603	-0.96948173	0.01600172
C	0.59111714	-1.35564261	-0.42067590
C	1.65285075	-0.37657080	0.08296747
H	-4.00035671	-0.63119495	-1.80208617
H	-3.19767581	-2.38181839	1.95579068
H	-5.75534277	-1.30858783	1.97809239
H	-6.45440846	-0.05772969	-1.54573432
H	-7.28934548	-0.62420923	-0.10396157
H	-6.40438512	1.35117704	1.18075124
H	-5.56225001	1.90630595	-0.26005981
H	-7.76353026	2.14089472	-1.44944043
H	-8.60134427	1.58388957	-0.01296038
H	-8.65743875	4.07919178	-0.14853332
H	-7.73577788	3.57620874	1.27144247
H	-6.89263194	4.13692137	-0.17569933
H	-1.65737835	-2.97408650	-0.13270752
H	-1.87128800	-2.00240775	-1.58509725

H	-1.07358950	0.02884964	-0.35675806
H	-0.87400928	-0.92259242	1.10851282
H	0.82287862	-2.36303945	-0.05446007
H	0.63452388	-1.40913895	-1.51514164
H	1.42412448	0.62988698	-0.28894047
H	1.60004710	-0.31650017	1.17718191
C	3.07241329	-0.76244598	-0.33507099
H	3.29760033	-1.77039265	0.03613531
H	3.12459966	-0.82372470	-1.42957997
C	4.13874883	0.21136910	0.16871143
H	4.08162661	0.27691013	1.26287398
H	3.91680468	1.21853782	-0.20685955
C	5.55990052	-0.17923613	-0.24011679
H	5.78076053	-1.18656642	0.13584882
H	5.61724275	-0.24557240	-1.33425821
C	6.62779476	0.79299472	0.26373817
H	6.40910998	1.79982514	-0.11491923
H	6.56804694	0.86175323	1.35765082
C	8.04928846	0.39893870	-0.14031945
H	8.10997806	0.32933149	-1.23427401
H	8.26859989	-0.60771293	0.23890945
C	9.11889454	1.37054809	0.36260540
H	9.05754210	1.44075800	1.45550907
H	8.90087145	2.37592708	-0.01790391
C	10.53587786	0.96564909	-0.04494868
H	10.63571228	0.91929332	-1.13401185
H	11.28043723	1.67566187	0.32578765
H	10.79379266	-0.02179239	0.35079817

Volume: 2992.83194 Bohr^3 (443.49194 Angstrom^3)

Estimated density according to mass and volume (M/V): 1.0465 g/cm^3

Minimal value: 16.12943 kcal/mol Maximal value: 123.91838 kcal/mol

Overall surface area: 1509.53933 Bohr^2 (422.71406 Angstrom^2)

Positive surface area: 1509.53933 Bohr^2 (422.71406 Angstrom^2)

Negative surface area: 0.00000 Bohr^2 (0.00000 Angstrom^2)

Overall average value: 0.09771822 a.u. (61.31916 kcal/mol)

Positive average value: 0.09771822 a.u. (61.31916 kcal/mol)

Negative average value: NaN a.u. (NaN kcal/mol)

Overall variance (sigma^2_tot): 0.00210121 a.u.^2 (827.39190 (kcal/mol)^2)

Positive variance: 0.00210121 a.u.^2 (827.39190 (kcal/mol)^2)

Negative variance: 0.00000000 a.u.^2 (0.00000 (kcal/mol)^2)

Balance of charges (nu): 0.00000000

Product of sigma^2_tot and nu: 0.00000000 a.u.^2 (0.00000 (kcal/mol)^2)

Internal charge separation (Pi): 0.04016539 a.u. (25.20418 kcal/mol)

Molecular polarity index (MPI): 2.65904805 eV (61.31916 kcal/mol)

Nonpolar surface area (|ESP| <= 10 kcal/mol): 0.00 Angstrom^2 (0.00 %)

Polar surface area (|ESP| > 10 kcal/mol): 422.71 Angstrom^2 (100.00 %)

$[BCIm]^+$

1 1

C	-7.18401360	-0.82565513	-0.67412852
N	-6.28849800	-1.49582301	0.05546410
C	-6.76992907	-1.61362427	1.34647531
C	-7.98387152	-1.00069434	1.37630309
N	-8.22623094	-0.51899442	0.10310446
C	-9.40115017	0.26771220	-0.30826030
C	-9.24771761	1.74415572	0.04821917
C	-10.47653709	2.55493288	-0.36792459
C	-10.33797091	4.03754765	-0.02288765
C	-4.97861396	-1.97409831	-0.41844850
C	-3.84207768	-1.06403040	0.04005369
C	-2.48291571	-1.57818574	-0.43637465
C	-1.32317236	-0.69673848	0.03009703
H	-7.08421754	-0.57861202	-1.71703050
H	-6.21829608	-2.12765525	2.11403192
H	-8.69363463	-0.87474804	2.17519658
H	-9.52277574	0.12877661	-1.38217220
H	-10.26818942	-0.17416634	0.18285392
H	-9.08557082	1.84196055	1.12662064
H	-8.35298371	2.13883197	-0.44450349
H	-10.63965751	2.44101768	-1.44554236
H	-11.36595656	2.14398995	0.12290755
H	-11.22459834	4.59900443	-0.32758318
H	-10.20584297	4.18217511	1.05341527
H	-9.47370789	4.48131836	-0.52577689
H	-4.85294686	-2.98952313	-0.04194164
H	-5.03158262	-2.02683812	-1.50546964

H	-4.01762212	-0.05304400	-0.34153012
H	-3.85036866	-0.99685416	1.13270841
H	-2.33153244	-2.60086330	-0.07057179
H	-2.47783429	-1.63942578	-1.53125395
H	-1.47382511	0.32621475	-0.33659141
H	-1.33595399	-0.63280367	1.12530987
C	0.04309996	-1.20608637	-0.43096501
H	0.18538449	-2.23408400	-0.07395435
H	0.05973386	-1.26063360	-1.52693896
C	1.20700049	-0.33899483	0.05145033
H	1.18543196	-0.28226713	1.14740090
H	1.06761545	0.68881947	-0.30754562
C	2.57540412	-0.85177811	-0.40002204
H	2.70931058	-1.88297710	-0.04845027
H	2.60134037	-0.90033970	-1.49629836
C	3.74092933	0.00524729	0.09643233
H	3.60985129	1.03622035	-0.25687460
H	3.71161844	0.05541000	1.19260816
C	5.11013218	-0.51150405	-0.34815161
H	5.14290012	-0.55548152	-1.44448295
H	5.23729685	-1.54487608	-0.00035972
C	6.27644699	0.33818588	0.15899373
H	6.24143054	0.38321743	1.25523783
H	6.15137357	1.37139795	-0.18998489
C	7.64600926	-0.18161731	-0.28093756
H	7.68338081	-0.22246770	-1.37725047
H	7.76848728	-1.21640632	0.06431812
C	8.81276005	0.66313840	0.23341808
H	8.77413292	0.70451381	1.32967073
H	8.69157042	1.69784836	-0.11245492
C	10.18249229	0.14142200	-0.20387667
H	10.22248168	0.10245058	-1.30017149
H	10.30226759	-0.89416711	0.13992647
C	11.34937045	0.98334807	0.31453081
H	11.30968364	1.02276994	1.41096618
H	11.23100229	2.01923443	-0.02927915
C	12.72010029	0.46153704	-0.12118020
H	12.76028988	0.42341417	-1.21665357

H	12.83793997	-0.57356589	0.22206791
C	13.87918273	1.30934851	0.40381695
H	13.88395739	1.33773937	1.49806038
H	14.84625648	0.91559124	0.07874189
H	13.80571096	2.34219551	0.04884451

Volume: 3792.45646 Bohr^3 (561.98407 Angstrom^3)

Estimated density according to mass and volume (M/V): 1.0330 g/cm^3

Minimal value: 10.41753 kcal/mol Maximal value: 123.80229 kcal/mol

Overall surface area: 1887.74399 Bohr^2 (528.62216 Angstrom^2)

Positive surface area: 1887.74399 Bohr^2 (528.62216 Angstrom^2)

Negative surface area: 0.00000 Bohr^2 (0.00000 Angstrom^2)

Overall average value: 0.08405617 a.u. (52.74609 kcal/mol)

Positive average value: 0.08405617 a.u. (52.74609 kcal/mol)

Negative average value: NaN a.u. (NaN kcal/mol)

Overall variance (sigma^2_tot): 0.00242470 a.u.^2 (954.76946 (kcal/mol)^2)

Positive variance: 0.00242470 a.u.^2 (954.76946 (kcal/mol)^2)

Negative variance: 0.00000000 a.u.^2 (0.00000 (kcal/mol)^2)

Balance of charges (nu): 0.00000000

Product of sigma^2_tot and nu: 0.00000000 a.u.^2 (0.00000 (kcal/mol)^2)

Internal charge separation (Pi): 0.04308632 a.u. (27.03710 kcal/mol)

Molecular polarity index (MPI): 2.28728480 eV (52.74609 kcal/mol)

Nonpolar surface area (|ESP| <= 10 kcal/mol): 0.00 Angstrom^2 (0.00 %)

Polar surface area (|ESP| > 10 kcal/mol): 528.62 Angstrom^2 (100.00 %)

[BOIm][Pd(CN)$_4$]$^-$

-1 1

C	0.49770357	2.23644047	-0.65906801
N	-0.72951308	2.70869356	-0.42853963
C	-0.65072238	3.69814502	0.53638339
C	0.65949966	3.80985381	0.88455220
N	1.36044447	2.89273656	0.12240466
C	2.79663376	2.58809226	0.23656685
C	3.06543262	1.59967471	1.36739839
C	4.53739553	1.19335196	1.43092270
C	4.79902508	0.15470152	2.52059868
C	-1.96369453	2.21344913	-1.06094579

C	-2.90939199	1.58843072	-0.04080442
C	-4.14756171	0.99897240	-0.71674704
C	-5.11184067	0.35535490	0.28074163
H	0.76050765	1.45976837	-1.35607746
H	-1.51903221	4.23436773	0.87635820
H	1.15155503	4.45538419	1.59090687
H	3.11101450	2.17950092	-0.72247273
H	3.31667111	3.53357401	0.39144885
H	2.75072162	2.03765843	2.32066012
H	2.44894538	0.70938957	1.20718736
H	4.83752715	0.78239340	0.46202997
H	5.15808560	2.08051055	1.60204058
H	5.85207494	-0.13741671	2.54484452
H	4.53636541	0.54158487	3.51006337
H	4.20849714	-0.74794483	2.34408255
H	-2.42828483	3.05062420	-1.58448107
H	-1.65777312	1.47582886	-1.80132673
H	-2.36738043	0.80324995	0.49049214
H	-3.21272237	2.34056822	0.69468420
H	-4.67182900	1.78188721	-1.27852561
H	-3.83418104	0.24917639	-1.45315496
H	-4.58329700	-0.42689027	0.83964487
H	-5.42061233	1.10327911	1.02186874
C	-6.35293396	-0.24494282	-0.38106749
H	-6.88207062	0.53885665	-0.93813755
H	-6.04242883	-0.98904326	-1.12565780
C	-7.31515214	-0.89618308	0.61340607
H	-7.62448248	-0.15298643	1.35982697
H	-6.78614301	-1.68097098	1.16955752
C	-8.55878852	-1.49647216	-0.04532851

H	-9.08816411	-0.71174045	-0.59945059
H	-8.24939721	-2.23803366	-0.79191396
C	-9.51264660	-2.14713293	0.95697952
H	-9.01988939	-2.95869034	1.50167256
H	-9.86396028	-1.42092047	1.69683024
Pd	2.47338726	-1.56822162	-0.49133215
C	2.61050914	-0.23792788	-1.99148410
N	2.65855563	0.58185923	-2.81541226
C	2.33034979	-2.86966958	1.03434251
N	2.25134555	-3.61136052	1.92590791
C	0.49705031	-1.19944196	-0.38597747
N	-0.64100920	-0.96420956	-0.34669463
C	4.44780194	-1.92874541	-0.61420674
N	5.58936749	-2.13389732	-0.69066303
H	-10.39248067	-2.56723084	0.46168249

[BUIm][Pd(CN)$_4$]$^-$

-1 1

C	1.97948442	2.27902443	-0.60707949
N	0.83774687	2.92845193	-0.36838393
C	1.05118742	3.84280889	0.64897124
C	2.35477726	3.72762115	1.01952234
N	2.91815728	2.74953337	0.21932119
C	4.28943284	2.22456224	0.33113224
C	4.41082602	1.21980636	1.47285063
C	5.81289614	0.61550869	1.54766152
C	5.92663490	-0.44123133	2.64543571
C	-0.44577703	2.66617426	-1.04092763
C	-1.49157740	2.12568694	-0.07119203
C	-2.78897680	1.76512821	-0.79532360
C	-3.86350856	1.23156149	0.15316849

H	2.13149466	1.50803221	-1.34254120
H	0.27180805	4.49237642	1.00666349
H	2.92702507	4.25204298	1.76486078
H	4.52931930	1.76169373	-0.62469529
H	4.95330672	3.07808222	0.47116553
H	4.15769621	1.70756466	2.42026294
H	3.67645107	0.42291789	1.31809807
H	6.05901499	0.16085563	0.58322812
H	6.54776114	1.41109143	1.71685365
H	6.93135326	-0.87060800	2.67881178
H	5.71237839	-0.01587590	3.63077287
H	5.22159464	-1.25832573	2.47198296
H	-0.77086240	3.59610259	-1.51020150
H	-0.24051989	1.94299022	-1.82901388
H	-1.07964761	1.23979499	0.41665043
H	-1.69610639	2.87013885	0.70523333
H	-3.17475126	2.64433718	-1.32600002
H	-2.57618357	1.01115967	-1.56295254
H	-3.47246414	0.35546955	0.68533877
H	-4.07549214	1.98531615	0.92188396
C	-5.16317914	0.85430825	-0.55891216
H	-5.54957346	1.72979444	-1.09641631
H	-4.95006063	0.09798705	-1.32511194
C	-6.24204672	0.32564845	0.38751561
H	-6.45505855	1.08286167	1.15309401
H	-5.85488884	-0.54874494	0.92629089
C	-7.54213197	-0.05361067	-0.32329905
H	-7.92706535	0.81998097	-0.86514777
H	-7.32946876	-0.81322758	-1.08657209
C	-8.62349986	-0.57732815	0.62320523

H	-8.23849002	-1.45039978	1.16581779
H	-8.83650625	0.18287067	1.38590160
C	-9.92328423	-0.95736118	-0.08762142
H	-9.71138155	-1.71915626	-0.84920482
H	-10.30805282	-0.08504058	-0.63197287
C	-11.00693155	-1.47867355	0.85839503
H	-11.21906844	-0.71706178	1.61871411
H	-10.62254428	-2.35023857	1.40220752
C	-12.30145431	-1.85586685	0.13740986
H	-12.12565168	-2.64017839	-0.60563267
H	-13.05880315	-2.22442282	0.83494276
H	-12.72617657	-0.99475946	-0.38824758
Pd	3.40907625	-1.72139287	-0.52682309
C	3.66381454	-0.42959856	-2.04526536
N	3.78639076	0.37212485	-2.87916208
C	3.15277151	-2.98740990	1.01356933
N	3.01248316	-3.71396300	1.91005322
C	1.49845617	-1.10968974	-0.36259688
N	0.39953081	-0.73789255	-0.28465846
C	5.31930511	-2.32306555	-0.70280535
N	6.42551892	-2.66473637	-0.80632538

$[BCIm][Pd(CN)_4]^-$

-1 1

C	4.49681155	2.23092540	-0.44814657
N	3.41572985	2.97760980	-0.21283311
C	3.67695306	3.81102239	0.86125011
C	4.94660934	3.54517307	1.27074009
N	5.44297679	2.55954907	0.43629532
C	6.74414120	1.88298435	0.57028797
C	6.68718665	0.77047698	1.61295768

C	8.00554694	0.00184388	1.69726370
C	7.93812457	-1.14427651	2.70561341
C	2.14362202	2.88388834	-0.94875780
C	0.99210037	2.47398947	-0.03707897
C	-0.30342642	2.28601397	-0.82690165
C	-1.48152115	1.88440976	0.06181319
H	4.60059572	1.48658378	-1.21824152
H	2.95112548	4.51530510	1.22737022
H	5.53643341	3.96765754	2.06548831
H	6.99652975	1.48586246	-0.41165491
H	7.48029010	2.64401390	0.82947774
H	6.43330889	1.19537555	2.58994486
H	5.87966100	0.08110988	1.34560056
H	8.24984908	-0.40040699	0.70918395
H	8.81629289	0.68895156	1.96604627
H	8.88140223	-1.69537340	2.74251538
H	7.72718621	-0.77464891	3.71385904
H	7.15051853	-1.85240932	2.43541149
H	1.96061612	3.84949469	-1.42310374
H	2.29403301	2.14121214	-1.73057799
H	1.26456240	1.53924782	0.45769095
H	0.84401825	3.23167283	0.73927873
H	-0.54930010	3.21167467	-1.36183547
H	-0.14968487	1.51829743	-1.59470123
H	-1.22909867	0.96317372	0.60152276
H	-1.63625499	2.65384115	0.82865813
C	-2.78113379	1.67575838	-0.71669326
H	-3.02912517	2.59531976	-1.26220870
H	-2.62580209	0.90290536	-1.48027522
C	-3.96247315	1.28020547	0.17055771

H	-4.11931213	2.05541074	0.93168543
H	-3.71229046	0.36336086	0.71969808
C	-5.26214403	1.06475456	-0.60647097
H	-5.50970890	1.97987989	-1.15984259
H	-5.10635203	0.28604048	-1.36422141
C	-6.44525544	0.67619540	0.28162407
H	-6.19661971	-0.23707749	0.83753638
H	-6.60240706	1.45664319	1.03739133
C	-7.74445247	0.45651009	-0.49508180
H	-7.58837291	-0.32675469	-1.24812260
H	-7.99113040	1.36840121	-1.05420073
C	-8.92871131	0.07371106	0.39401650
H	-9.08573407	0.85819560	1.14562764
H	-8.68140619	-0.83690942	0.95488642
C	-10.22754326	-0.14872978	-0.38253388
H	-10.07140094	-0.93531691	-1.13209896
H	-10.47340526	0.76085705	-0.94574382
Pd	5.39208931	-1.92829645	-0.57594786
C	5.96643797	-0.58088306	-1.95258813
N	6.27773369	0.25347435	-2.70097764
C	4.81796983	-3.24654085	0.82901779
N	4.48911307	-3.99847228	1.65220775
C	3.57772069	-1.05757355	-0.52494259
N	2.53898271	-0.53507538	-0.51776523
C	7.20384780	-2.79693391	-0.64864743
N	8.25108873	-3.29900692	-0.69622233
C	-11.41257459	-0.52715647	0.50742710
H	-11.56929255	0.26019983	1.25606760
H	-11.16638742	-1.43594836	1.07172466
C	-12.71123996	-0.75124402	-0.26902479

H	-12.55515019	-1.53999576	-1.01632881
H	-12.95654812	0.15690304	-0.83478978
C	-13.89661975	-1.12680083	0.62152753
H	-14.05380587	-0.33788504	1.36864393
H	-13.65196227	-2.03475747	1.18812926
C	-15.19619271	-1.35205171	-0.15399740
H	-15.03940734	-2.14112539	-0.89958209
H	-15.44045411	-0.44495968	-0.72026861
C	-16.37448312	-1.72576171	0.74595121
H	-16.57605171	-0.93911074	1.47993385
H	-17.28969163	-1.88157649	0.16795590
H	-16.17155290	-2.64770967	1.30004029

[BUIm][Pd(CN)$_4$][BUIm]

0 1

C	1.93564400	-3.77434300	0.89577900
N	0.73576800	-4.32676100	1.08930200
C	0.61028700	-5.42177000	0.25200600
C	1.76971100	-5.51745700	-0.45283300
N	2.58612300	-4.48301300	-0.03171000
C	3.91099500	-4.14836300	-0.58085100
C	3.79483300	-3.37672900	-1.89169800
C	5.16454600	-2.95509800	-2.42356300
C	5.05529900	-2.13328500	-3.70702400
C	-0.30176800	-3.81157300	1.99830900
C	-1.50931600	-3.28359200	1.23075200
C	-2.54896200	-2.67028700	2.16911100
C	-3.73395800	-2.07195200	1.41046800
H	2.32658500	-2.90701000	1.39991100
H	-0.27511000	-6.03280900	0.24077800
H	2.08763500	-6.22423600	-1.19921100

H	4.42600900	-3.55831800	0.17541800
H	4.45458100	-5.08471100	-0.70953700
H	3.27280800	-3.98936600	-2.63441600
H	3.17897200	-2.48778000	-1.72174300
H	5.67927900	-2.36422400	-1.65945800
H	5.78052100	-3.84443300	-2.60016000
H	6.04174400	-1.83380000	-4.07050400
H	4.56501700	-2.70260500	-4.50273700
H	4.47361100	-1.22381300	-3.53682100
H	-0.57377600	-4.61810600	2.68093700
H	0.16324500	-3.01843200	2.58275600
H	-1.15906100	-2.53037500	0.52216600
H	-1.96504600	-4.09645000	0.65582800
H	-2.90834100	-3.42995900	2.87418100
H	-2.07578800	-1.88756400	2.77461700
H	-3.36856700	-1.28815800	0.73569100
H	-4.17991500	-2.84124200	0.76745400
C	-4.81525300	-1.49211800	2.32333300
H	-5.20655200	-2.28618000	2.97226700
H	-4.36759400	-0.74575600	2.99211700
C	-5.96510500	-0.85247800	1.54551700
H	-6.37521600	-1.58527900	0.83884600
H	-5.57138400	-0.03537300	0.93092200
C	-7.09576500	-0.32230600	2.42767800
H	-7.49679800	-1.14226200	3.03758700
H	-6.69371800	0.41381200	3.13586800
C	-8.23074000	0.31301000	1.62285700
H	-7.83114800	1.14462000	1.02928700
H	-8.60922700	-0.41643800	0.89594600
C	-9.39087700	0.81849300	2.48006700

H	-9.01691400	1.55331900	3.20500200
H	-9.79525900	-0.01242600	3.07286400
C	-10.51851800	1.44776100	1.65894300
H	-10.88795400	0.71347100	0.93263900
H	-10.11368700	2.27862400	1.06820400
C	-11.68044600	1.94838700	2.51725800
H	-11.34606400	2.70868000	3.23024000
H	-12.47108100	2.39269700	1.90612300
H	-12.12657000	1.13153600	3.09339200
Pd	3.49283500	0.00341200	-0.49306700
C	4.06565500	-0.99751600	1.15111800
N	4.35810800	-1.63486700	2.07898700
C	2.91791400	0.97708500	-2.15594900
N	2.61104700	1.56589800	-3.11029600
C	1.57907700	-0.43513400	-0.05895500
N	0.47804600	-0.69639100	0.20681600
C	5.40682500	0.44541800	-0.92685300
N	6.50400800	0.73551400	-1.17878900
C	4.06965000	3.59174700	-0.14124500
N	2.89622500	4.21391900	0.00352300
C	2.87932800	4.84473300	1.23449500
C	4.07643000	4.59073300	1.82895300
N	4.80637700	3.80910900	0.95189400
C	6.13540000	3.23071400	1.21379400
C	6.06015100	2.10362100	2.23923600
C	7.41569600	1.42001100	2.42216300
C	7.36090700	0.29915300	3.46056400
C	1.77597400	4.15852000	-0.95189600
C	0.64402600	3.27589200	-0.43525600
C	-0.46719300	3.13064300	-1.47472600

C	-1.64683000	2.29978200	-0.96675000
H	4.36785500	3.00439300	-0.99091400
H	2.03376500	5.42064300	1.56749300
H	4.47380500	4.90191800	2.77909700
H	6.51035800	2.85676200	0.26301900
H	6.78516300	4.04066000	1.54745700
H	5.70696600	2.49792100	3.19825000
H	5.32225300	1.37122500	1.90212100
H	7.73740600	1.01477500	1.45738400
H	8.16713200	2.16262900	2.71553300
H	8.33209100	-0.19201500	3.56513300
H	7.07722200	0.68573400	4.44449900
H	6.62698500	-0.45951100	3.17690800
H	1.44329000	5.18259200	-1.12575100
H	2.17441900	3.76224900	-1.88378900
H	1.04886800	2.29162000	-0.18792500
H	0.23854300	3.69931800	0.48999500
H	-0.82427800	4.12378200	-1.77533600
H	-0.05064700	2.66500700	-2.37479700
H	-1.28511900	1.31471700	-0.65146800
H	-2.06373500	2.77569100	-0.06968900
C	-2.75295500	2.13421100	-2.01000100
H	-3.07506600	3.12482900	-2.35671500
H	-2.34615000	1.62294100	-2.89200600
C	-3.96969700	1.36554000	-1.49313800
H	-4.36743800	1.87540800	-0.60609700
H	-3.65657200	0.37069500	-1.15230800
C	-5.08388500	1.22067700	-2.53185500
H	-5.36612600	2.21625600	-2.89854200
H	-4.70002400	0.67521000	-3.40368000

C	-6.32702500	0.51264000	-1.99276900
H	-6.04974100	-0.48296000	-1.62587000
H	-6.69969000	1.05817400	-1.11786500
C	-7.45472800	0.37843700	-3.01623300
H	-7.09644900	-0.19172300	-3.88341400
H	-7.72109400	1.37301200	-3.39758600
C	-8.70691300	-0.29465500	-2.44951400
H	-9.06699600	0.28117300	-1.58895100
H	-8.43908800	-1.28368400	-2.05791100
C	-9.83021000	-0.43719600	-3.47658600
H	-9.50980100	-1.03958000	-4.33258100
H	-10.71240400	-0.91828500	-3.04484000
H	-10.14050200	0.53984400	-3.86053800

Volume: 7078.77150 Bohr^3 (1048.96571 Angstrom^3)

Estimated density according to mass and volume (M/V): 1.2181 g/cm^3

Minimal value: -68.15831 kcal/mol Maximal value: 52.18837 kcal/mol

Overall surface area: 2956.55296 Bohr^2 (827.91915 Angstrom^2)

Positive surface area: 1661.99069 Bohr^2 (465.40479 Angstrom^2)

Negative surface area: 1294.56227 Bohr^2 (362.51436 Angstrom^2)

Overall average value: -0.00412655 a.u. (-2.58945 kcal/mol)

Positive average value: 0.02125944 a.u. (13.34051 kcal/mol)

Negative average value: -0.03671770 a.u. (-23.04072 kcal/mol)

Overall variance (sigma^2_tot): 0.00151746 a.u.^2 (597.52859 (kcal/mol)^2)

Positive variance: 0.00045877 a.u.^2 (180.65116 (kcal/mol)^2)

Negative variance: 0.00105869 a.u.^2 (416.87743 (kcal/mol)^2)

Balance of charges (nu): 0.21092680

Product of sigma^2_tot and nu: 0.00032007 a.u.^2 (126.03479 (kcal/mol)^2)

Internal charge separation (Pi): 0.02886254 a.u. (18.11153 kcal/mol)

Molecular polarity index (MPI): 0.76268128 eV (17.58786 kcal/mol)

Nonpolar surface area (|ESP| <= 10 kcal/mol): 412.15 Angstrom^2 (49.78 %)

Polar surface area (|ESP| > 10 kcal/mol): 415.77 Angstrom^2 (50.22 %)

$[BOIm]^+$- n-heptane-H_2O

1 1

C	2.45794220	-0.96990070	0.94975062
N	1.79446950	-2.10837661	1.18070377
C	2.67118435	-3.17081806	1.06032188
C	3.88911260	-2.64698567	0.75421811
N	3.73426007	-1.27364063	0.69494431
C	4.77826037	-0.30443108	0.32176089
C	4.86638463	-0.11996704	-1.19073253
C	6.00081661	0.83269203	-1.57231490
C	6.06027227	1.09557497	-3.07668897
C	0.35139138	-2.18872249	1.46385301
C	-0.48901872	-1.88999823	0.22536309
C	-1.98377968	-1.90477829	0.54599377
C	-2.84919389	-1.48373583	-0.64198773
H	2.02115461	0.02549131	0.92641023
H	2.35623496	-4.18854071	1.21157017
H	4.84093026	-3.11946801	0.58492776
H	4.53938369	0.64144983	0.80507114
H	5.71667097	-0.67401581	0.73543179
H	5.01556187	-1.09238174	-1.67199578
H	3.91048019	0.27280914	-1.55325127
H	5.86843482	1.77739161	-1.03601865
H	6.95524123	0.41476710	-1.23218595
H	6.87941071	1.77382281	-3.32877710
H	6.21250719	0.16767786	-3.63613395
H	5.13098778	1.54903695	-3.43445727
H	0.15897955	-3.19005668	1.84691917
H	0.13502313	-1.48202245	2.26585269

H	-0.20507355	-0.90917209	-0.16758787
H	-0.25855692	-2.62355991	-0.55361246
H	-2.27892268	-2.90537263	0.88341327
H	-2.18052944	-1.22897144	1.38677119
H	-2.53574115	-0.48738678	-0.97481405
H	-2.66754649	-2.15794648	-1.48796384
C	-4.34292407	-1.45940122	-0.31632188
H	-4.67680850	-2.47147838	-0.05519920
H	-4.50644863	-0.84633642	0.57781667
C	-5.20287395	-0.91620643	-1.45768293
H	-5.06610483	-1.54095081	-2.34981111
H	-4.84668885	0.08514630	-1.73263534
C	-4.73241934	2.82704068	1.44444883
H	-4.84021000	3.69113677	0.78137862
H	-4.62749619	3.20645139	2.46571029
C	-3.53127626	1.97141975	1.04519013
H	-3.46853898	1.09765454	1.70393408
H	-3.68476968	1.57629638	0.03486289
C	-2.20350947	2.73030803	1.09066045
H	-2.04123452	3.11898900	2.10395409
H	-2.26523415	3.60724140	0.43388675
C	-1.01489681	1.86356226	0.67498172
H	-1.17480804	1.47703876	-0.33754588
H	-0.94387550	0.99246615	1.33563742
C	0.30965883	2.60330203	0.69783163
H	0.52929097	2.95840796	1.71423258
H	0.25603538	3.48542935	0.04559880
O	1.33773011	1.72402349	0.24213607
H	2.18157174	2.21752721	0.19679403
C	-6.69139874	-0.84050495	-1.11327809

H	-7.05061563	-1.83932572	-0.83704097
H	-6.82165408	-0.21419791	-0.22217789
O	3.77271796	2.93914372	0.19516136
H	4.00755809	3.40445076	-0.61529616
H	3.98916497	3.52580313	0.92835458
C	-7.54342258	-0.28725654	-2.25579203
H	-7.45784986	-0.91142198	-3.15089593
H	-8.60164241	-0.24160318	-1.98384350
H	-7.22760288	0.72448789	-2.52930954
H	-5.66321925	2.25485927	1.39665893

Volume: 3641.14671 Bohr^3 (539.56227 Angstrom^3)

Estimated density according to mass and volume (M/V): 1.0574 g/cm^3

Minimal value: 22.13011 kcal/mol Maximal value: 122.90798 kcal/mol

Overall surface area: 1689.36072 Bohr^2 (473.06918 Angstrom^2)

Positive surface area: 1689.36072 Bohr^2 (473.06918 Angstrom^2)

Negative surface area: 0.00000 Bohr^2 (0.00000 Angstrom^2)

Overall average value: 0.09619066 a.u. (60.36060 kcal/mol)

Positive average value: 0.09619066 a.u. (60.36060 kcal/mol)

Negative average value: NaN a.u. (NaN kcal/mol)

Overall variance (sigma^2_tot): 0.00121946 a.u.^2 (480.18514 (kcal/mol)^2)

Positive variance: 0.00121946 a.u.^2 (480.18514 (kcal/mol)^2)

Negative variance: 0.00000000 a.u.^2 (0.00000 (kcal/mol)^2)

Balance of charges (nu): 0.00000000

Product of sigma^2_tot and nu: 0.00000000 a.u.^2 (0.00000 (kcal/mol)^2)

Internal charge separation (Pi): 0.02962653 a.u. (18.59095 kcal/mol)

Molecular polarity index (MPI): 2.61748110 eV (60.36060 kcal/mol)

Nonpolar surface area (|ESP| <= 10 kcal/mol): 0.00 Angstrom^2 (0.00 %)

Polar surface area (|ESP| > 10 kcal/mol): 473.07 Angstrom^2 (100.00 %)

$[BUIm]^{+}$- n-heptane-H_2O

1 1

C	-3.64873980	-1.34724484	-0.55772889
N	-2.88815184	-2.44430846	-0.62726632
C	-3.52769864	-3.47787853	0.03327690
C	-4.70459366	-2.97928310	0.50078445
N	-4.76102055	-1.65116517	0.11965111
C	-5.83126018	-0.69188124	0.44776529
C	-5.36481895	0.35160860	1.45903947
C	-6.49940858	1.29483713	1.86182425
C	-6.03592546	2.36789906	2.84714023
C	-1.54316385	-2.48970633	-1.22167133
C	-0.45345556	-2.27470335	-0.17382636
C	0.92531583	-2.12633185	-0.81662859
C	2.02530749	-1.83762756	0.20584839
H	-3.40750467	-0.36346070	-0.95231471
H	-3.09866339	-4.46214691	0.10266003
H	-5.50093213	-3.44574926	1.05395027
H	-6.15432199	-0.22023481	-0.48056665
H	-6.66500100	-1.27715904	0.83363275
H	-4.96456710	-0.15741783	2.34213123
H	-4.54578293	0.92670821	1.01695622
H	-6.90533394	1.76959083	0.96332515
H	-7.31673035	0.71536539	2.30598271
H	-6.85495790	3.03872059	3.11839865
H	-5.65234498	1.92064259	3.76898737
H	-5.23412648	2.97776427	2.41944671
H	-1.43795300	-3.45162618	-1.72324084
H	-1.50944724	-1.71217714	-1.98456844
H	-0.68930228	-1.37058111	0.39647769
H	-0.45751598	-3.10720417	0.53700096

H	1.17328846	-3.03148205	-1.38343118
H	0.89101526	-1.30753305	-1.54453110
H	1.71754030	-0.98985156	0.82952151
H	2.13075775	-2.69053274	0.88723925
C	3.37508119	-1.51972903	-0.43886107
H	3.73883356	-2.40007793	-0.98343998
H	3.23613522	-0.73458790	-1.19145903
C	4.43360466	-1.06249484	0.56512308
H	4.59575861	-1.84927263	1.31289546
H	4.05144539	-0.19404824	1.11704119
C	2.84434411	2.64476662	-0.70248647
H	2.79445525	3.23901676	0.21525972
H	2.94924094	3.34064434	-1.54070687
C	1.60290850	1.76855777	-0.86072055
H	1.69690282	1.15940560	-1.76711241
H	1.54847699	1.06128377	-0.02679349
C	0.29779102	2.56366586	-0.92537883
H	0.34004578	3.26541638	-1.76784552
H	0.20258254	3.17798511	-0.02105208
C	-0.93251224	1.66637262	-1.06625272
H	-0.97858753	0.96859772	-0.22255460
H	-0.84668237	1.05608390	-1.97295086
C	-2.23587914	2.44280275	-1.12914572
H	-2.20755317	3.15639575	-1.96387195
H	-2.37530814	3.02035124	-0.20549317
O	-3.31422918	1.52528615	-1.30667631
H	-4.16361784	2.01228118	-1.28706451
C	5.76752025	-0.69492902	-0.08598290
H	6.15936192	-1.56380960	-0.63017800
H	5.59717312	0.08297681	-0.84159431

O	-5.69769504	2.82053545	-1.11132591
H	-5.68231975	3.49022000	-0.41867441
H	-6.06082594	3.24343281	-1.89747576
C	6.81789502	-0.20510728	0.91180950
H	6.99411088	-0.98368341	1.66520869
H	6.42118999	0.66040246	1.45817361
C	8.14686838	0.17683886	0.25840361
H	7.97013006	0.95604848	-0.49450751
H	8.54449968	-0.68758113	-0.28923378
C	9.19832870	0.66906226	1.25493783
H	9.37685734	-0.11034770	2.00576222
H	8.80006768	1.53168441	1.80304515
C	10.52116128	1.05187118	0.59052426
H	11.25477769	1.39924354	1.32338075
H	10.95905032	0.19924795	0.06188237
H	10.37829152	1.85360224	-0.14090976
H	3.75496997	2.04100087	-0.65622752

Volume: 4131.37327 Bohr^3 (612.20636 Angstrom^3)

Estimated density according to mass and volume (M/V): 1.0460 g/cm^3

Minimal value: 16.04317 kcal/mol Maximal value: 118.21914 kcal/mol

Overall surface area: 1889.09026 Bohr^2 (528.99915 Angstrom^2)

Positive surface area: 1889.09026 Bohr^2 (528.99915 Angstrom^2)

Negative surface area: 0.00000 Bohr^2 (0.00000 Angstrom^2)

Overall average value: 0.09025850 a.u. (56.63811 kcal/mol)

Positive average value: 0.09025850 a.u. (56.63811 kcal/mol)

Negative average value: NaN a.u. (NaN kcal/mol)

Overall variance (sigma^2_tot): 0.00131935 a.u.^2 (519.51750 (kcal/mol)^2)

Positive variance: 0.00131935 a.u.^2 (519.51750 (kcal/mol)^2)

Negative variance: 0.00000000 a.u.^2 (0.00000 (kcal/mol)^2)

Balance of charges (nu): 0.00000000

Product of sigma^2_tot and nu: 0.00000000 a.u.^2 (0.00000 (kcal/mol)^2)

Internal charge separation (Pi): 0.03079479 a.u. (19.32404 kcal/mol)

Molecular polarity index (MPI): 2.45605867 eV (56.63811 kcal/mol)

Nonpolar surface area (|ESP| <= 10 kcal/mol): 0.00 Angstrom^2 (0.00 %)

Polar surface area (|ESP| > 10 kcal/mol): 529.00 Angstrom^2 (100.00 %)

$[BCIm]^{+}$- n-heptane-H_2O

1 1

C	-6.05743503	-1.09261326	-0.35033603
N	-5.49599998	-2.30579534	-0.33733970
C	-6.28509618	-3.15814864	0.41342618
C	-7.34883575	-2.43006283	0.85017440
N	-7.18895442	-1.14670026	0.35975542
C	-8.06930110	0.00617475	0.62382770
C	-7.45965864	0.96324816	1.64460269
C	-8.41884035	2.10548795	1.98365738
C	-7.78856987	3.13185668	2.92473451
C	-4.19372104	-2.62759378	-0.94215336
C	-3.03906266	-2.30685921	0.00368397
C	-1.68041916	-2.50882919	-0.66673074
C	-0.51664975	-2.10152180	0.23779551
H	-5.65348705	-0.19678458	-0.81344964
H	-6.02678138	-4.19272035	0.55774146
H	-8.19845236	-2.70863047	1.44885411
H	-8.24597206	0.51599473	-0.32315040
H	-9.01937027	-0.39925658	0.97046913
H	-7.19782153	0.40820014	2.55162829
H	-6.53091754	1.36784461	1.23144837
H	-8.72942829	2.59718405	1.05675619
H	-9.32817599	1.69581159	2.43810394

H	-8.48873249	3.93721464	3.16095292
H	-7.48182857	2.67027163	3.86818379
H	-6.90025145	3.58466796	2.47394991
H	-4.21465127	-3.68322437	-1.21126457
H	-4.11705825	-2.05260723	-1.86521720
H	-3.13203494	-1.26643160	0.33068777
H	-3.11966584	-2.92935183	0.90051380
H	-1.56611157	-3.55499853	-0.97431455
H	-1.64339119	-1.91290347	-1.58664621
H	-0.68902289	-1.08033611	0.59577301
H	-0.50295428	-2.73820623	1.13076009
C	0.84216189	-2.16328507	-0.46072828
H	1.07168171	-3.20199061	-0.72967480
H	0.78526202	-1.61185484	-1.40696611
C	1.98040788	-1.58554500	0.38089483
H	2.06426795	-2.14615508	1.32067841
H	1.72802979	-0.55583210	0.66414784
C	1.05502450	1.99545427	-1.59948884
H	1.20873228	2.92452781	-1.04170174
H	1.08635537	2.24174894	-2.66544157
C	-0.27036641	1.33619677	-1.22210006
H	-0.37799410	0.39326958	-1.76913031
H	-0.25442036	1.06784260	-0.16007335
C	-1.49173844	2.21495019	-1.49706856
H	-1.50655495	2.49483417	-2.55802578
H	-1.39820343	3.15309866	-0.93557053
C	-2.80775471	1.52692558	-1.13116550
H	-2.79643607	1.23836026	-0.07385671
H	-2.91290690	0.60044554	-1.70758541
C	-4.02778258	2.39488708	-1.38337333

H	-4.07341955	2.67987916	-2.44344101
H	-3.95982459	3.32001686	-0.79533681
O	-5.19649963	1.66492015	-1.01663953
H	-5.99440298	2.21694821	-1.14976196
C	3.32985352	-1.59051285	-0.33802672
H	3.59690685	-2.62092497	-0.60566294
H	3.23414272	-1.04694492	-1.28697830
O	-7.51531094	3.05794449	-1.22566512
H	-7.52539066	3.87112152	-0.70867808
H	-7.86120363	3.27711075	-2.09795693
C	4.45972907	-0.97021393	0.48487417
H	4.55479429	-1.50943257	1.43624134
H	4.19045246	0.06109981	0.74739463
C	5.80948189	-0.97198114	-0.23435296
H	5.71261377	-0.43616837	-1.18751987
H	6.08059500	-2.00355102	-0.49378217
C	6.93777070	-0.34398688	0.58536634
H	7.03270526	-0.87771498	1.53988347
H	6.66749168	0.68849003	0.84213315
C	8.28848103	-0.34892228	-0.13225645
H	8.55944703	-1.38166429	-0.38719443
H	8.19325648	0.18308184	-1.08770947
C	9.41615007	0.28153606	0.68649703
H	9.14611388	1.31498428	0.93951537
H	9.50991668	-0.24902382	1.64288284
C	10.76763611	0.27362677	-0.02976514
H	10.67386839	0.80337872	-0.98661311
H	11.03802452	-0.75998134	-0.28180420
C	11.89487361	0.90510988	0.78862713
H	11.62581519	1.93938973	1.03994046

H	11.98864515	0.37640203	1.74620896
C	13.24771493	0.89614912	0.07392719
H	13.15410036	1.42461156	-0.88268245
H	13.51675648	-0.13732263	-0.17644620
C	14.36695826	1.52981873	0.90075821
H	15.32198079	1.51061301	0.36829076
H	14.14055888	2.57427886	1.13745621
H	14.50567451	1.00083150	1.84897878
H	1.90177211	1.33693337	-1.38621869

Volume: 4927.02110 Bohr^3 (730.10920 Angstrom^3)

Estimated density according to mass and volume (M/V): 1.0366 g/cm^3

Minimal value: 10.47754 kcal/mol Maximal value: 119.27905 kcal/mol

Overall surface area: 2267.60015 Bohr^2 (634.99272 Angstrom^2)

Positive surface area: 2267.60015 Bohr^2 (634.99272 Angstrom^2)

Negative surface area: 0.00000 Bohr^2 (0.00000 Angstrom^2)

Overall average value: 0.07971610 a.u. (50.02265 kcal/mol)

Positive average value: 0.07971610 a.u. (50.02265 kcal/mol)

Negative average value: NaN a.u. (NaN kcal/mol)

Overall variance (sigma^2_tot): 0.00161737 a.u.^2 (636.87017 (kcal/mol)^2)

Positive variance: 0.00161737 a.u.^2 636.87017 (kcal/mol)^2)

Negative variance: 0.00000000 a.u.^2 (0.00000 (kcal/mol)^2)

Balance of charges (nu): 0.00000000

Product of sigma^2_tot and nu: 0.00000000 a.u.^2 (0.00000 (kcal/mol)^2)

Internal charge separation (Pi): 0.03457213 a.u. (21.69436 kcal/mol)

Molecular polarity index (MPI): 2.16918535 eV (50.02265 kcal/mol)

Nonpolar surface area (|ESP| <= 10 kcal/mol): 0.00 Angstrom^2 (0.00 %)

Polar surface area (|ESP| > 10 kcal/mol): 634.99 Angstrom^2 (100.00 %)

H_2O

Volume: 181.51759 Bohr^3 (26.89813 Angstrom^3)

Estimated density according to mass and volume (M/V): 1.1122 g/cm^3

Minimal value: -43.81841 kcal/mol Maximal value: 44.56807 kcal/mol

Overall surface area: 156.50588 Bohr^2 (43.82611 Angstrom^2)

Positive surface area: 79.49797 Bohr^2 (22.26170 Angstrom^2)

Negative surface area: 77.00791 Bohr^2 (21.56441 Angstrom^2)

Overall average value: 0.00114324 a.u. (0.71740 kcal/mol)

Positive average value: 0.04106661 a.u. (25.76971 kcal/mol)

Negative average value: -0.04007106 a.u. (-25.14499 kcal/mol)

Overall variance (sigma^2_tot): 0.00083319 a.u.^2 (328.08574 (kcal/mol)^2)

Positive variance: 0.00042270 a.u.^2 (166.44560 (kcal/mol)^2)

Negative variance: 0.00041050 a.u.^2 (161.64014 (kcal/mol)^2)

Balance of charges (nu): 0.24994637

Product of sigma^2_tot and nu: 0.00020825 a.u.^2 (82.00384 (kcal/mol)^2)

Internal charge separation (Pi): 0.04056605 a.u. (25.45560 kcal/mol)

Molecular polarity index (MPI): 1.10414961 eV (25.46232 kcal/mol)

Nonpolar surface area (|ESP| <= 10 kcal/mol): 7.23 Angstrom^2 (16.49 %)

Polar surface area (|ESP| > 10 kcal/mol): 36.60 Angstrom^2 (83.51 %)

n-heptane

Volume: 1254.91357 Bohr^3 (185.95901 Angstrom^3)

Estimated density according to mass and volume (M/V): 0.8948 g/cm^3

Minimal value: -3.63638 kcal/mol Maximal value: 6.84263 kcal/mol

Overall surface area: 683.28700 Bohr^2 (191.33985 Angstrom^2)

Positive surface area: 487.44077 Bohr^2 (136.49732 Angstrom^2)

Negative surface area: 195.84623 Bohr^2 (54.84253 Angstrom^2)

Overall average value: 0.00280367 a.u. (1.75933 kcal/mol)

Positive average value: 0.00507366 a.u. (3.18377 kcal/mol)

Negative average value: -0.00284610 a.u. (-1.78595 kcal/mol)

Overall variance (sigma^2_tot): 0.00000959 a.u.^2 (3.77639 (kcal/mol)^2)

Positive variance: 0.00000711 a.u.^2 (2.80056 (kcal/mol)^2)

Negative variance: 0.00000248 a.u.^2 (0.97583 (kcal/mol)^2)

Balance of charges (nu): 0.19163073

Product of sigma^2_tot and nu: 0.00000184 a.u.^2 (0.72367 (kcal/mol)^2)

Internal charge separation (Pi): 0.00366307 a.u. (2.29861 kcal/mol)

Molecular polarity index (MPI): 0.12068766 eV (2.78313 kcal/mol)

Nonpolar surface area (|ESP| <= 10 kcal/mol): 191.34 Angstrom^2 (100.00 %)

Polar surface area (|ESP| > 10 kcal/mol): 0.00 Angstrom^2 (0.00 %)
